# pH-Thermo Dual-Responsive Polymeric Nanoparticles for Women’s Health: Dual Action Against Cervical and Ovarian Cancer Cells

**DOI:** 10.1021/acsami.5c18234

**Published:** 2025-10-28

**Authors:** Giuseppe Nunziata, Emanuele Limiti, Dania Aramini, Marco Nava, Luca Moretti, Alberto Rainer, Mattia Sponchioni, Filippo Rossi

**Affiliations:** † Department of Chemistry, Materials and Chemical Engineering “Giulio Natta”, 18981 Politecnico di Milano, Piazza Leonardo da Vinci 32, 20133 Milan, Italy; ‡ Department of Science and Technology for Sustainable Development and One Health, Università Campus Bio-Medico di Roma, via Álvaro del Portillo 21, 00128 Rome, Italy; § CNR NANOTEC, Istituto di Nanotecnologia, Via Monteroni, 73100 Lecce, Italy; ∥ Department of Science and Engineering of Matter, Environment and Urban Planning, Università Politecnica delle Marche, via Brecce Bianche 12, 60131 Ancona, Italy; ⊥ Department of Engineering, Università Campus Bio-Medico di Roma, via Álvaro del Portillo 21, 00128 Rome, Italy

**Keywords:** colloids, drug delivery, nanoparticles, pH-responsive, thermoresponsive, cancer, polymers

## Abstract

The development of smart nanocarriers capable of responding to tumor-specific stimuli represents a promising strategy for improving therapeutic selectivity in oncology. In this work, we present a class of dual-responsive polymeric nanoparticles (NPs) engineered for precision drug delivery in gynecological cancers. Amphiphilic block copolymers of the type P­(MAA)-*b*-P­(EG_2_MA-*co*-NIPAM) integrating pH-responsive methacrylic acid (MAA) and thermoresponsive diethylene glycol methyl ether methacrylate (EG_2_MA) and *N*-isopropylacrylamide (NIPAM) units were synthesized via reversible addition–fragmentation chain transfer (RAFT) polymerization. Fine-tuning of the lower critical solution temperature (LCST) was achieved by modulating the ratio between NIPAM and EG_2_MA, yielding copolymers with cloud points within the physiologically relevant range of 30–40 °C. The resulting NPs exhibited sharp and reversible swelling/shrinking behavior in response to pH and temperature stimuli, with sizes below 182 nm and narrow polydispersity indexes. The core–shell architecture was stabilized by a dodecyl-functionalized chain transfer agent, ensuring efficient self-assembly and robust encapsulation of both hydrophilic and hydrophobic drugs. Drug release studies with 5-fluorouracil (5-FU) and the drug-mimetic fluorescein isothiocyanate (FITC) confirmed a marked temperature-triggered release above the LCST and enhanced diffusion in mildly acidic conditions (pH < 6), characteristic of solid tumors. Cellular studies on HeLa and ovarian adenocarcinoma OVCA433 lines revealed rapid internalization, high biocompatibility, and a significant increase in therapeutic efficacy of 5-FU when delivered via NPs, compared to the free drug. These findings highlight the potential of the dual-responsive nanoplatform for targeted and controlled delivery in the treatment of cervical and ovarian cancers.

## Graphical abstract

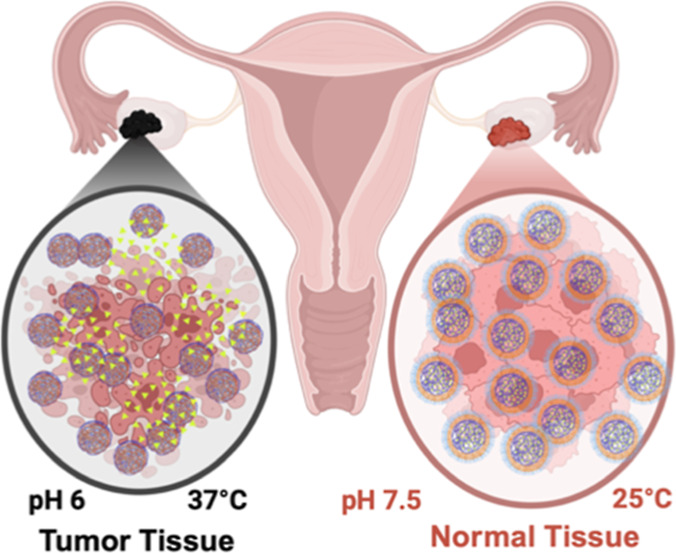

## Introduction

Gynaecological cancers remain a critical health burden worldwide, accounting for a substantial proportion of cancer-related morbidity and mortality among women.^1^ These malignancies, primarily affecting the ovaries, cervix, and endometrium, are often diagnosed at advanced stages due to the lack of early and specific symptoms, combined with suboptimal screening programs.^2^ Among them, ovarian and cervical cancers are particularly aggressive and represent a major clinical challenge, with poor prognostic outcomes once metastasis occurs.^3^ In recent years, it has become increasingly evident that the tumor microenvironment in gynecologic cancers is characterized by distinct physicochemical hallmarks, including local acidosis and elevated temperature associated with inflammation and rapid metabolic turnover.^4–6^ These features provide a unique opportunity for the development of responsive drug delivery systems capable of selectively releasing therapeutic agents in situ, thereby maximizing therapeutic efficacy while minimizing systemic toxicity.^7^ In recent years, stimuli-responsive nanoparticles (NPs) have attracted growing interest in cancer therapy.^8–10^ Most often, they are based on amphiphilic polymers which self-assemble into micelle structures with a hydrophobic core and a hydrophilic shell once dispersed in water.^11,12^ Various classes of responsive NPs have been developed, each tailored to react to specific stimuli, including changes in pH, temperature or magnetic field.^13,14^ In drug delivery applications, the sensitivity to these physical stimuli can be advantageously exploited to achieve more selective treatments, enabling drug release only in environments characterized by specific local conditions.^15^ Indeed, stimuli-responsive NPs are capable of systemic circulation and can release their cargo only upon reaching diseased tissues, such as tumors, which often present altered pH and temperature profiles.^16,17^ Methacrylic acid (MAA) and *N*-isopropylacrylamide (NIPAM) are among the most common monomers utilized for the synthesis of stimuli-responsive polymers.^18–20^ The former is known to be pH-responsive due to the reversible protonation and deprotonation of its carboxylic group across its p*K*_a_, which leads to changes in molecular interactions and nanoparticle structure.^21^

Holappa and co-workers, for example, studied the pH-responsiveness of double-hydrophilic block copolymers of linear PEO–PMAA, evidencing the formation of aggregates at pH 4.5 and macroscopic precipitation below pH 3.8.^22^ This effect is due to protonation of the carboxylic acids and the consequent increase in hydrogen bonding interactions among MAA units. A similar effect was observed for the NPs developed by Liu and co-workers, based on PMAA–PEO-PMAA triblock copolymers.^23^ Micelles formed via a self-assembly mechanism in the pH range of 2–6, where the low solubility of MAA in its protonated form promoted aggregation and the formation of a hydrophobic core, whereas no micelle formation was observed at pH > 6 due to complete polymer solubility. Aggregation was evidenced by an increase in hydrodynamic radius at pH < 2.6. In the field of thermoresponsive polymers, NIPAM is well-known for its lower critical solution temperature (LCST) of 32 °C. Below this threshold temperature, NIPAM is hydrophilic and water-soluble; however, when the temperature is exceeded, the polymer undergoes a coil-to-globule phase transition and becomes hydrophobic.^24,25^ Due to the proximity of its LCST to the physiological temperature, NIPAM has been extensively studied for biomedical applications. In addition, its copolymerization with hydrophilic or hydrophobic monomers allows fine-tuning of the LCST.^26,27^ Copolymerization of PNIPAM with hydrophilic monomers such as PEG-based methacrylates in a random fashion typically leads to an increase in the overall LCST, as the incorporation of hydrophilic units reduces the polymer tendency to collapse at lower temperatures. As a result, micelle formation is observed only above this adjusted LCST, where the polymer becomes sufficiently amphiphilic to promote self-assembly.^28^ Conversely, copolymerization with hydrophobic monomers results in PNIPAM forming the outer shell of the micelle, which collapses above LCST, enabling a more sustained and controlled drug release.^29,30^ In addition, both methacrylic acid-based and PNIPAM-containing polymers are well-known to exhibit moderate mucoadhesive properties, due to hydrogen bonding and hydrophobic interactions with mucin glycoproteins, especially under acidic conditions typical of the cervicovaginal environment.^31–33^

Beyond PNIPAM-based systems, another promising class of thermoresponsive materials is represented by poly­(oligo­(ethylene glycol) methacrylate)­s (POEGMAs), which LCST behavior can be finely tuned by varying both the polymer chain length and the number of ethylene glycol units per side chain.^24^ Systematic investigations have shown that the homopolymers of OEGMA monomers exhibit temperature-responsive behavior strongly dependent on the degree of polymerization and the side chain length. For instance, a decrease in the number of ethylene glycol units or substitution of the methoxy end group with a more hydrophobic ethoxy group can significantly lower the cloud point due to increased hydrophobicity.^34^ Dual-responsiveness to both pH and temperature can be intriguingly accessed by copolymerizing hydrophilic monomers bearing carboxylic groups, such as acrylic acid or methacrylic acid, with NIPAM.^35,36^ The resulting double-hydrophilic block copolymers can self-assemble depending on block fraction, mutual interactions, and external stimuli.^37^ Still, their systematic investigation and possibility of application in oncology is in its infancy. To bridge this gap, the present work introduces dual pH- and thermoresponsive nanoparticles as an evolution of a previously reported pH-responsive system designed by our group.^38^ Building on that foundation, the formulation was re-engineered to introduce thermo-responsivity and achieve more refined control over drug release. Specifically, double-hydrophilic block copolymers were synthesized in which a strongly hydrophobic chain transfer agent (CTA) constitutes the inner core, while two sequential hydrophilic blocks, first PMAA and then a copolymer of NIPAM-*co*-EG_2_MA, form the two outer shells. The use of the hydrophobic CTA, covalently preserved at the chain end after the RAFT process, during the reversible addition–fragmentation chain transfer (RAFT) polymerization was a critical design element to drive the self-assembly of the amphiphilic constructs into core–shell nanoparticles in aqueous environments, while ensuring efficient encapsulation of hydrophobic therapeutic agents.^39–41^ RAFT polymerization enabled precise control over molecular weight and low polydispersity. Different ratios of NIPAM and EG_2_MA were tested to modulate the LCST in the range 30–40 °C, suitable for biomedical applications.

Cloud point values were determined via UV–vis spectroscopy, which reflects the bulk phase transition of the polymer, whereas DLS provided insight into the apparent LCST, defined as the temperature at which nanoparticle size undergoes a sharp change due to chain collapse and aggregation. These transition values were further validated through drug release studies conducted above and below the apparent LCST. To assess the therapeutic potential of the developed nanocarriers, the chemotherapeutic drug 5-fluorouracil (5-FU) was used as a model anticancer agent. The nanoparticles demonstrated efficient loading of 5-FU and triggered release in response to thermal stimuli. Additionally, all formulations displayed marked pH-responsiveness below pH 6, attributable to the presence of MAA units, which undergo protonation under mild acidic conditions, mimicking the acidic tumor microenvironment. Their biological performance was further evaluated on two gynecological cancer cell lines, OVCA433 (ovarian carcinoma) and HeLa (cervical carcinoma), to investigate biocompatibility, cellular uptake, and therapeutic efficacy. The dual responsiveness of the polymeric system (Figure 1), combined with tunable LCST and hydrophobic core design, resulted in a promising platform for selective drug delivery in tumor environments, supporting the potential application of these smart nanocarriers in precision oncology.

**Figure 1:**
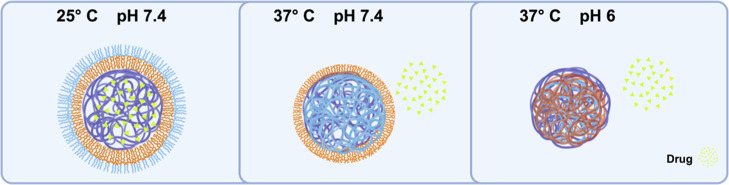
Schematic representation of the dual pH- and thermoresponsive behavior of the nanoparticles in different physiological environments. In the first panel (25 °C, pH 7.4), the NP is shown with a core of C_12_ from CTA (purple) surrounded by a hydrophobic shell composed of the pH-responsive polymer PMAA in orange. An additional outer shell, formed by a thermoresponsive polymer NIPAM-*co*-EG_2_MA (blue), stabilizes the nanoparticle in aqueous environments. In the second panel (37 °C, pH 7.4), due to the increased temperature, the thermoresponsive block undergoes a phase transition, collapsing around the nanoparticle core. This leads to a partial release of the encapsulated drug (green triangles), while the PMAA layer remains stable at neutral pH. In the third panel (37 °C, pH 6), the acidic pH triggers the protonation of the PMAA layer, leading to further destabilization of the nanoparticle structure. This results in the full collapse of the nanoparticle and a complete release of the encapsulated drug (green).

## Materials and Methods

### Materials

2,2′-Azobis­(2-methylpropionitrile) (AIBN, Sigma-Aldrich); 4-cyano-4-[(dodecyl sulfanylthiocarbonyl)­sulfany]­pentanoic acid (Chain Transfer Agent, CTA, Sigma-Aldrich); 4-cyano-4-(phenylcarbonothioylthio)-pentanoic acid (Sigma-Aldrich); hydroxyethyl methacrylate (HEMA, Sigma-Aldrich); methacrylic acid (MAA, Sigma-Aldrich); di­(ethylene glycol) methyl ether methacrylate (EG_2_MA); *N*-isopropylacrylamide (NIPAM, Sigma-Aldrich); *N*,*N*′-dicyclohexylcarbodiimide (DCC, Sigma-Aldrich); 4-(dimethylamino)­pyridine (DMAP, Sigma-Aldrich); ethanolamine (Sigma-Aldrich); Dulbecco’s phosphate buffered saline (Sigma-Aldrich); 5-fluorouracil (5-FU, Sigma-Aldrich); fluorescein isothiocyanate isomer I (FITC, Sigma-Aldrich); rhodamine B (RhB, Sigma-Aldrich); acetone (Sigma-Aldrich); acetonitrile (ACN, Sigma-Aldrich); Toluene (Sigma-Aldrich); ethanol (Sigma-Aldrich); dichloromethane (DCM, Sigma-Aldrich); dichloromethane anhydrous (Sigma-Aldrich); dimethyl sulfoxide (DMSO, Sigma-Aldrich); hydrochloric acid (HCl, Sigma-Aldrich); sodium chloride (NaCl, Sigma-Aldrich); sodium hydroxide (NaOH; Sigma-Aldrich); sodium bicarbonate (NaHCO_3_, Sigma-Aldrich); ethyl acetate (Sigma-Aldrich) were of analytical grade purity and used as received.

### Synthesis of PMAA

PMAA was synthesized via RAFT polymerization in hermetically sealed Pyrex vials (10 mL) put in a block heater. MAA (0.295 g, 3.45 mmol) and CTA (0.028 g, 0.069 mmol) were added and dissolved in 5 mL of ACN under magnetic stirring. Once a homogeneous solution was obtained, the initiator AIBN (0.004 g, 0.023 mmol) was mixed in. To ensure an inert reaction environment, a flow of nitrogen was introduced into the mixture for 30 min. The system was shielded from light and then heated up to 70 °C; the reaction proceeded for 24 h under constant stirring.

Samples were collected at the initial and final time points, dried under a gentle stream of nitrogen, and subsequently analyzed by proton nuclear magnetic resonance (^1^H NMR) to assess monomer conversion.

### Synthesis of (PMAA)-*b*-P­(EG_2_MA-*co*-NIPAM)

(PMAA)-*b*-P­(EG_2_MA-*co*-NIPAM) was synthesized via a second RAFT polymerization in hermetically sealed Pyrex vials (10 mL) put in a block heater. The PMAA macromolecular chain transfer agent previously synthesized, EG_2_MA and NIPAM were mixed under magnetic stirring in 5 mL of ACN. Once a homogeneous solution was obtained, the initiator AIBN (0.004 g, 0.023 mmol) was mixed in. To ensure an inert reaction environment, a flow of nitrogen was introduced into the mixture for 30 min. The system was shielded from light and then heated up to 70 °C; the reaction proceeded for 24 h under constant stirring. Samples were collected at the initial and final time points, dried under a gentle stream of nitrogen, and subsequently analyzed by proton nuclear magnetic resonance (^1^H NMR) to assess monomer conversion. Different formulations were synthesized, to study how the amount and ratio between the thermoresponsive monomers affected the polymer characteristics. The recipes for the different block copolymers are provided in Table 1.

**Table 1:** EG_2_MA and NIPAM Quantities for Four Different (PMAA)-*b*-P­(EG_2_MA-*co*-NIPAM) Formulations

			EG_2_MA		NIPAM	
#	sample	EG_2_MA/NIPAM	g	mmol	g	mmol
A	(MAA)_50_-*b*-(EG_2_MA_17_-*co*-NIPAM_33_)	17/33	0.216	1.15	0.260	2.3
B	(MAA)_50_-*b*-(EG_2_MA_25_-*co*-NIPAM_25_)	25/25	0.325	1.73	0.195	1.73
C	(MAA)_50_-*b*-(EG_2_MA_46_-*co*-NIPAM_4_)	46/4	0.597	3.17	0.031	0.28
D	(MAA)_50_-*b*-(EG_2_MA_50_-*co*-NIPAM_50_)	50/50	0.649	3.45	0.390	3.45

### Nanoparticle Production

Nanoparticles were obtained by precipitating the (PMAA)-*b*-P­(EG_2_MA-*co*-NIPAM) block copolymers in distilled water. Initially, 50 mg of polymer were dissolved in 1 mL of ethanol. Nanoprecipitation was then performed by dripping the solution with a 20–200 μL pipet into a 25 mL vial, containing 10 mL of distilled water under stirring at 600 rpm. The resulting particle suspension was stirred for at least 30 min. To study drug encapsulation and release, FITC and 5-FU were used. In particular, the same protocol of the previous section was carried out, by replacing the volume of ethanol with a 2 mg/mL solution of the drug in ethanol. Three mL of NP suspension was dialyzed for 30 min, by using 3.5 kDa cellulose membranes against 40 mL PBS. Each system was produced in triplicate (A, B, C) and studied in parallel. For all the systems, efficiency and drug loading were calculated according to eqs 1 and 2, respectively.

1 $$\%\;Eff=\left(1-\frac{C_{v}}{C_{0}}\right)\times 100$$

2 $$\%\;DL=\left(\frac{m_{ed}}{m_{ed}+m_{p}}\right)\times 100$$

In eq 1, *C*_0_ is the total drug concentration in the external medium (15 ppm) and *C*_v_ is the drug concentration outside the membrane after 30 min of dialysis. In eq 2, *m*_ed_ is the mass of encapsulated drug and *m*_p_ is the mass of polymer. After washing, the dialysis external volume was replaced with fresh 40 mL of PBS. To assess the temperature influence on the release, a triplicate of a releasing system was kept at a temperature above the LCST of the polymer, another triplicate was maintained at 20 °C. To study the drug release over time, the volume outside the membrane was sampled after 1 h, 2 h, 4 h, 6 h, 24 h, 48 h, 72 h, 96 h, 7 d, 14 d, 21 d, and 28 d. Two mL of medium were collected and replaced with an equal volume of fresh PBS. 5-FU release samples were analyzed using high performance liquid chromatography (HPLC). The optimal parameters for the separation of 5-FU were selected using a mobile phase consisting of 60% aqueous solution (2% acetic acid and 98% distilled water) and 40% acetonitrile (ACN), with a flow rate of 1 mL/min.

The analysis was performed using a Roc C18 5 μm column (250 × 4.6 mm), at a temperature of 37 °C, with a detection wavelength of 266 nm and an injection volume of 10 μL. FITC-containing samples were analyzed via UV–vis spectrometry. One mL of sample were loaded in disposable plastic cuvettes. The wavelength for FITC was set to 495 nm. Absorbance was evaluated and sample concentration was calculated by the Beer–Lambert law, after a proper calibration. To further elucidate the release mechanism, the cumulative release data were analyzed according to the Korsmeyer–Peppas semiempirical model, which correlates the fractional drug release (*Q*_t_/*Q*_∞_) with time (h) through the equation *Q*_t_/*Q*_∞_ = *k*·*t* ^*n*^, where *n* is the release exponent and *k* a kinetic constant.^42^

### Polymers and Nanoparticle Characterization

The molecular weight distribution of the polymers and their precursors was analyzed using gel permeation chromatography (GPC). GPC was performed using a Jasco LC-2000 Plus chromatograph equipped with a refractive index detector (RI-2031 Plus, Jasco) using 3 Agilent PLgel columns, 5 × 10^–6^ M particle size, 300 × 7.5 mm (MW range: 5 × 10^12^ to 17 × 10^5^ g mol^–1^). Samples were injected using a Jasco AS-2055 Plus autosampler. The polymers were dissolved in DMAC to obtain a 4 mg/mL solution. Three drops of HCl were added to the sample, to enhance the solubility of the copolymers. The solutions were filtered with a 0.2 μm PTFE filter and loaded into a Scintillation Vial. Polystyrene standards were employed for calibrating the system, while a sample of solvent (DMAC + HCl) was injected for a blank run. NPs size, charge and their evolution based on pH and temperature variations were analyzed via DLS on a Zetasizer Nano ZS (Malvern Instruments) at a scattering angle of 173°. Samples were prepared by adding 100 μL of NPs suspension in 2.9 mL of PBS or buffers at different pH (1, 2, 3, 4, 5, 6, 8, 10, 12). A volume of 1 mL of the test solution was loaded in glass cuvettes. The refractive index was 1.590 and the absorption was set to 0.010.

Atomic force microscopy (AFM) and transmission electron microscopy (TEM) were employed to investigate the surface topography and internal morphology of the samples at the nanoscale. AFM analysis was performed using an NT-MDT Solver PRO instrument operating in tapping mode. A high-resolution NSG10 tip, with a nominal radius of 5–10 nm, was used to scan a 2 × 2 μm area of the sample surface. This technique enabled the acquisition of precise surface profiles and provided valuable information on the size and distribution of the nanoscale features. TEM analysis was conducted using an EFTEM Leo 912AB transmission electron microscope (Karl Zeiss, Jena, Germany) operating at 80 kV. For sample preparation, a 5 μL drop of nanoparticle dispersion was deposited onto a Formvar/carbon-coated copper grid and allowed to dry overnight at room temperature. Digital images were acquired using a charge-coupled device (CCD; Esi Vision Proscan camera), enabling high-resolution visualization of the internal structure and morphology of the nanoparticles. Optical transmittance measurements of the nanoparticles in aqueous solution under varying pH conditions were performed using a Jasco V-630 UV–vis spectrophotometer equipped with a custom-built thermostatic cell holder, allowing precise temperature control (±0.1 °C). To evaluate pH-responsiveness, samples were prepared by mixing 100 μL of NP suspension with 2.9 mL of buffer solutions at different pH values. Subsequently, 1 mL of each resulting solution was transferred into high performance quartz glass for analysis. To assess temperature-responsiveness, the same procedure was applied using PBS instead of buffered solutions. Transmittance was recorded to monitor changes in the optical properties of the nanoparticle dispersions in response to external stimuli. The critical micelle concentration (CMC) of the polymeric systems was determined using a Jasco FP8500 spectrofluorometer and pyrene as a fluorescent probe. A defined amount of pyrene, initially dissolved in acetone, was added to a series of clean glass vials. After complete evaporation of the solvent, polymer solutions at varying concentrations (ranging from 5000 mg/L to 0.001 mg/L) were introduced into each vial, ensuring a final pyrene concentration of 6 × 10^–7^ M. The samples were incubated in the dark at room temperature for 24 h to allow equilibration.

Fluorescence measurements were performed by exciting the samples at 335 nm and recording the emission spectra in the range of 350–450 nm. The slit widths were set to 5 nm for excitation and 2 nm for emission. The ratio of the third (*I*_3_, 384 nm) to the first (*I*_1_, 373 nm) vibronic band intensities in the pyrene emission spectrum (*I*_3_/*I*_1_) was used as an indicator of the local polarity and employed to determine the onset of micelle formation.

### Synthesis of HEMA-RhB and Formulation of RhB-Labeled NPs

The synthesis of HEMA-RhB was carried out following a previously reported protocol.^43^ RhB (1 g, 2.09 mmol) and HEMA (0.375 g, 2.88 mmol) were dissolved in 20 mL of ACN under magnetic stirring. Once the reagents were completely dissolved, a solution of DMAP (13 mg, 106.40 mmol) and DCC (0.43 g, 2.08 mmol) in ACN (20 mL) was then dripped slowly into the mixture, previously quenched in an ice bath. To ensure an inert reaction environment, a flow of nitrogen was introduced into the solution for 30 min. The reaction, shielded from light, proceeded for 24 h under constant magnetic stirring. Samples were collected at the initial and final time, to assess monomer conversion by an NMR analysis. ACN was evaporated under reduced pressure. The obtained solid product was redispersed in ethyl acetate (40 mL) and sodium bicarbonate (40 mL). The water phase was isolated and washed three times with fresh ethyl acetate (40 mL) and finally saturated with NaCl. The product was extracted with a mixture of DCM/IPA 1:2 v/v. The organic phase was extracted and desiccated with anhydrous sodium sulfate; the solvent was subsequently evaporated under reduced pressure. The stock solution for the following reactions was created by dissolving the final solid product in ACN. The copolymer P­((HEMA-RhB)-*graft*-MAA_49_)-*b*-(EG_2_MA_17_-*co*-NIPAM_33_) was formulated via flash nanoprecipitation, following the same optimized conditions used for the corresponding nonfluorescent nanoparticles. Nanoparticle formation was verified by dynamic light scattering (DLS).

To evaluate dye retention, dialysis experiments were performed: 3 mL of RhB-labeled NP suspension were placed into 3.5 kDa MWCO cellulose membranes and dialyzed against 40 mL of ultrapure water under continuous stirring. Aliquots of the external dialysis medium were collected at scheduled time points over 1 week, lyophilized, redissolved in 2 mL of water, and analyzed by UV–vis spectroscopy at λ = 555 nm.

### Cell Culture

HeLa cells, derived from human epithelial cervical carcinoma, and OVCA433 cells, derived from ovarian carcinoma, were cultured in Dulbecco’s modified Eagle’s medium–high glucose (DMEM high glucose, Thermo Fisher Scientific) supplemented with 10% fetal bovine serum (FBS), 100 U/mL penicillin–streptomycin, and 1 mM l-glutamine. Cells were incubated at 37 °C in a humidified atmosphere with 5% CO_2_.

### In Vitro Cytocompatibility

NP cytocompatibility of selected NP formulations (A and D) was assessed with HeLa and OVCA433 cells. via MTT assay (Merck KGaA, Darmstadt, Germany) Cells were seeded at a density of 5 × 10^3^ cells per well in a 96-well plate and cultured in a humidified incubator (5% CO_2_ at 37 °C) in complete medium for 24 h. Successively, cells were administered with different concentrations of NPs for 24 h. After incubation with the NPs, the culture medium was replenished with 100 μL of MTT solution (0.5 mg/mL) and incubated for 3 h at 37 °C. After incubation, medium was removed and 100 μL of DMSO were added to each well to solubilize the resulting formazan crystals. Finally, absorbance of the resulting solutions was evaluated by a spectrophotometer analysis (570 nm, TECAN Infinite M200-Pro) and the outcome values were normalized to those of untreated cells, which represent the negative control group.

### Nanoparticle Internalization

NP internalization by HeLa and OVCA433 cells was studied by flow cytometric and confocal microscopy analyses. For flow cytometry, cells were seeded at a density of 1.5 × 10^4^ cells/cm^2^ into 12-well plates for 24 h. The cells were washed in PBS and incubated for 45 min at room temperature and the NPs were administered at a final concentration of 0.1 mg/mL. At 2, 6, and 24 h time points, the cells were analyzed by flow cytometry (CytoFLEX flow cytometer, Beckman Coulter, Brea, CA) with CytExpert software (Beckman Coulter). The NPs signal was recorded in the allophycocyanin (PE-A) channel and quantified by the mean fluorescence intensity (MFI). Using confocal microscopy, both cell lines were seeded at a concentration of 1.5 × 10^4^ cells/cm^2^ into an 8-well chamber slide 24 h. After 24 h, the cells were fixed in paraformaldehyde (4% in PBS) for 15 min at room temperature and then incubated for 5 min in Triton X-100 (0.1% in PBS) to permeabilize the cell membranes. Subsequently, they were washed three times in PBS, incubated with ActinGreen 488 stain (1:80 dilution in PBS for 40 min, in the dark), washed in PBS (3 times), and counterstained with DAPI (1:1000 dilution in PBS for 10 min, in the dark). Micrographs were collected using a Nikon A1R + laser scanning confocal microscope with a 20× NA 0.7 air objective.

### Evaluation of Anticancer Efficacy

To study the effects induced by different administration methods of a specific drug dose, four groups were identified: (i) untreated cells (negative control group); (ii) cells treated with 5-FU at a concentration of 2.6 μg/mL; (iii) cells treated with unloaded NPs at a concentration of 0.1 mg/mL; and (iv) cells treated with 5-FU-loaded NPs with final drug and NP concentration of 2.6 μg/mL and 0.1 mg/mL, respectively. Experiments were performed on both HeLa and OVCA433 cell models. Cells were seeded at a density of 5 × 10^3^ cells per well in a 96-well plate and incubated for 24h prior to drug/NP administration according to the above-described experimental groups.

At each time point (24 h, 48 h, 72 h and 7 days), MTT assay was performed as previously described to measure cell viability. For each experimental group, viability levels were normalized against those of the negative control group and reported as mean value ± standard deviation.

### Statistical Analysis

The experimental data were analyzed using analysis of variance (ANOVA). Statistical significance was set to *p* value < 0.05. Results are presented as mean value ± standard deviation.

## Results and Discussion

### Polymer Synthesis and Characterization

He synthesis of the final amphiphilic block copolymer, whose structure is schematically represented in Figure 2, was carried out through a two-step RAFT polymerization approach.

**Figure 2:**
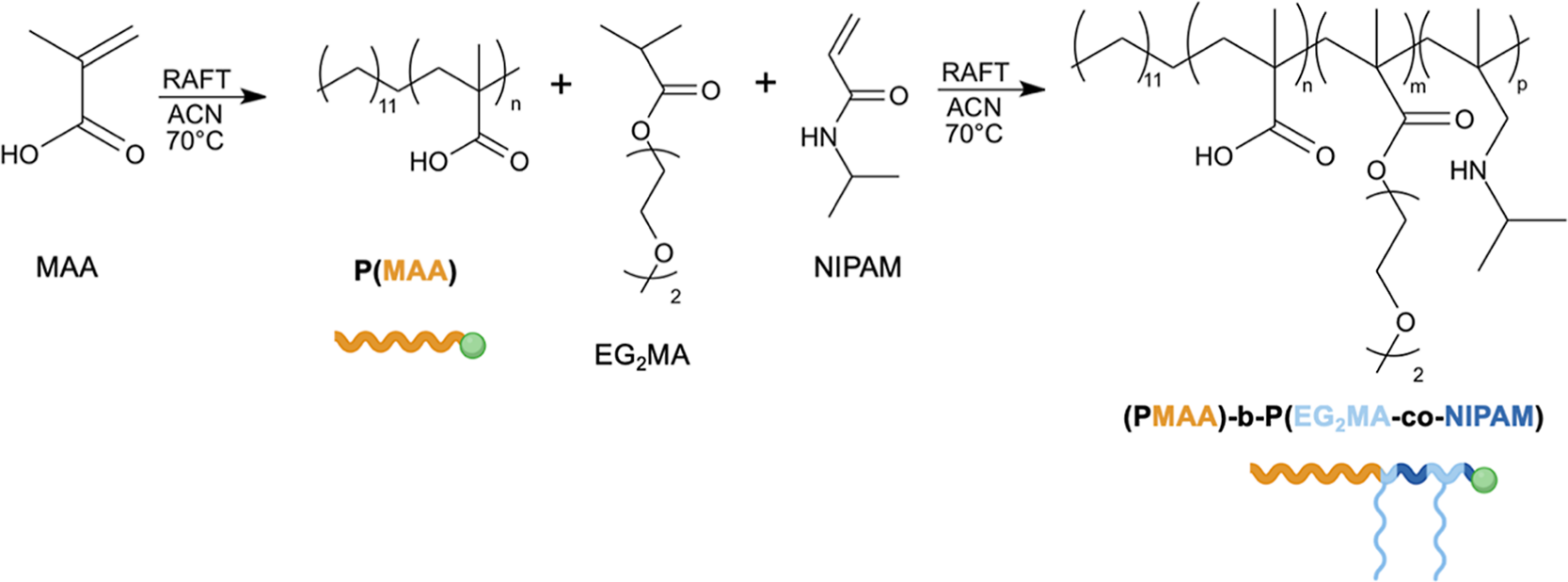
Scheme of polymerization reactions performed sequentially for the synthesis of (PMAA)-*b*-P­(EG_2_MA-*co*-NIPAM) copolymers.

First, a macromolecular chain transfer agent (macro-CTA) was synthesized via RAFT polymerization of MAA. To ensure reproducibility and evaluate kinetic control, RAFT polymerization was independently repeated several times for each formulation. The successful MAA polymerization was confirmed using ^1^H NMR spectroscopy (DMSO-*d*_6_, 400 MHz). To carry out the ^1^H NMR analysis, a fixed amount of TSP was added to each sample as an internal standard. The TSP peak, located at a chemical shift of 0 ppm, was used as a stable reference for calculating monomer conversion, as its intensity remains constant throughout the reaction.^44^ As polymerization proceeded, the formation of polymer chains led to an increase in the corresponding polymer peaks, while the intensity of the MAA monomer peak at 6.1 ppm progressively decreased relative to the TSP signal, reflecting the gradual consumption of the monomer (Figure S1). Figure 3a shows the monomer conversion profiles for MAA in six independent polymerizations. The results are reported as average with error bars showing the standard deviation.

**Figure 3:**
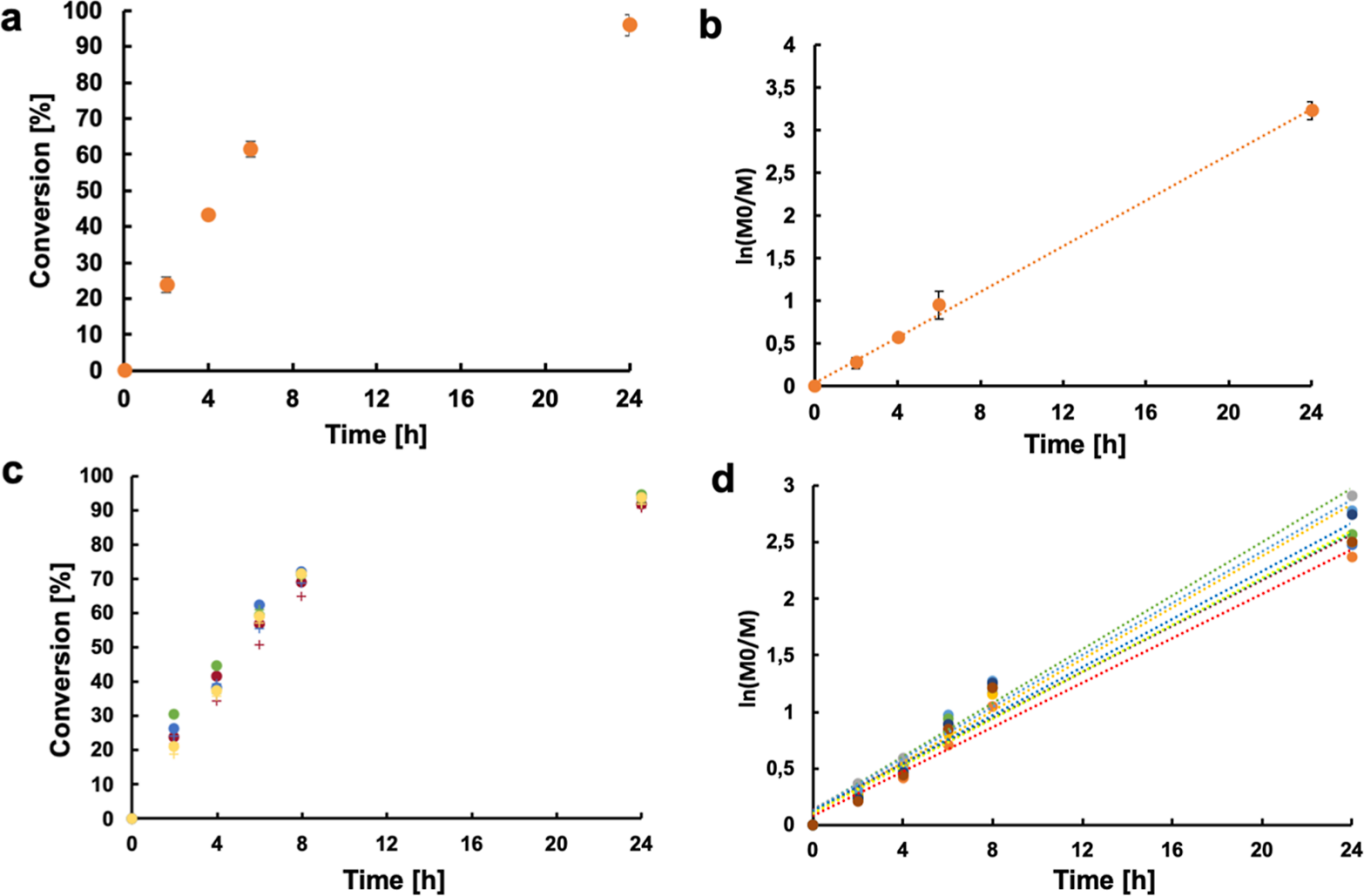
(a,b) Conversion and semilogarithmic plot of monomer conversion over time for the RAFT polymerization of MAA (circle). (c,d) Conversion and semilogarithmic plot of monomer conversions over time for the RAFT polymerization of NIPAM (cross) and EG_2_MA (circle) mediated by the PMAA macromolecular chain transfer agent for sample A (red) sample B (green) sample C (blue) sample D (yellow).

The corresponding semilogarithmic plots (Figure 3b) display linear trends, indicating a constant radical concentration and minimal termination events, typical features of a controlled RAFT process. The produced macro-CTA was then chain-extended with EG_2_MA and NIPAM in a second RAFT polymerization step to obtain four different amphiphilic block copolymers of the type (PMAA)-*b*-P­(EG_2_MA-*co*-NIPAM). These copolymers differ in the composition and relative length of the two functional blocks: the pH-responsive PMAA and the thermoresponsive block composed of EG_2_MA and NIPAM. By systematically varying the degree of polymerization (DP) of the latter, a small library of amphiphilic copolymers was obtained, each capable of self-assembling in aqueous solution into dual pH- and thermoresponsive nanoparticles. Monomer conversion data for this second step are shown in Figure 3c, with the semilogarithmic plots in Figure 3d again confirming linearity and the preservation of the living character of the RAFT polymerization.

The successful synthesis and control over the targeted block lengths were further confirmed by ^1^H NMR spectroscopy and GPC, whose results are reported in Table 2. All polymers showed low dispersity values (<1.22), confirming the high degree of control typical of RAFT polymerization.^45^ These results collectively confirm that the synthetic strategy allowed for robust and reproducible control over molecular structure and composition across the different formulations.

**Table 2:** Average MW and DP of Polymers Calculated by ^1^H NMR and GPC Analyses

		GPC			^1^H NMR		
#	sample	*M*_n_ (Da)	*M*_w_ (Da)	*D̵* (−)	*M*_n_ (Da)	DP pH-respectively	DP thermo-respectively
A	P(MAA)_50_-*b*-(EG_2_MA_17_-*co*-NIPAM_33_)	28,605	32,610	1.14	31,939	48.5	45.8
B	P(MAA)_50_-*b*-(EG_2_MA_25_-*co*-NIPAM_25_)	32,881	38,142	1.16	34,734	48.5	47.3
C	P(MAA)_50_-*b*-(EG_2_MA_46_-*co*-NIPAM_4_)	45,453	50,453	1.11	38,882	48.5	46.9
D	P(MAA)_50_-*b*-(EG_2_MA_50_-*co*-NIPAM_50_)	47,480	57,926	1.22	55,530	48.5	93.6

### Nanoparticles Structure

Although the P­(MAA) block plays a structural role in forming the core of P­(MAA)_50_-*b*-(EG_2_MA-*co*-NIPAM) NPs, it does not provide sufficient hydrophobicity to ensure core stability on its own, due to the presence of hydrophilic carboxylic groups.

However, based on further observations and control experiments, it is more accurate to suggest that the core is predominantly stabilized by the hydrophobic C_12_ alkyl chains introduced via the RAFT CTA, while the P­(MAA) block may act as an interfacial corona between the hydrophobic core and the thermoresponsive shell, especially at neutral pH. Indeed, several studies in the literature have demonstrated that long alkyl chains from CTAs, such as dodecyl groups, can enhance the stability, compactness, and self-assembly of polymeric nanoparticles by reinforcing the hydrophobic character of the core-forming block.^46–48^ In this paragraph, the aim was to verify that the C_12_ tail of the CTA used in our formulation plays a similar stabilizing role in the P­(MAA)_50_-*b*-(EG_2_MA-*co*-NIPAM) nanoparticles. First, several tests were conducted to demonstrate the absence of cross-links, which could lead to nanogels (NGs) formation. The formation of cross-links could be explained by the presence of dimethacrylate impurities in the commercial monomers, mainly EG_2_MA, as demonstrated in the literature.^49^ GPC and DLS analysis were performed to assess the absence of cross-links. The GPC analysis revealed a unimodal and narrow molecular weight distribution (Figure S2), indicating a highly controlled polymerization and excluding chain branching. This is further confirmed by NMR analysis. However, GPC analysis is not enough on itself to prove the absence of chemical cross-linking reactions: whether the side reactions were performed with a unitary conversion, a single pick could still be noticed. Therefore, further DLS analyses were carried out, with the aim of verifying the absence of chemical cross-links. P­(MAA)_50_-*b*-(EG_2_MA_50_-*co*-NIPAM_50_) was dissolved in ACN and acetone and the resulting solution was analyzed. The presence of branching would make a portion of the chains insoluble, leading to nanostructures detectable by DLS. However, the results obtained in terms of size and polydispersity did not show the presence of nanostructures. To further confirm the absence of physical nanogels, the polymer was nanoprecipitated in water, and the resulting nanoparticle suspension was dispersed in acetone and in a solution of EtOH 30%. Once again, the size and polydispersity values do not correspond to physical nanogels.

Physical cross-links should have formed through hydrogen bonding interactions at the interface between water and the organic solvent. Once the hypothesis of nanogel formation was ruled out, attention was turned to the influence of the chain transfer agent (CTA) on the self-assembly process. To this end, three PMAA-based polymers were synthesized under different conditions: one using a highly hydrophobic CTA containing a dodecyl chain (4-cyano-4-[(dodecylsulfanylthiocarbonyl)­sulfanyl]­pentanoic acid), one employing a less hydrophobic CTA bearing a phenyl group (4-cyano-4-(phenylcarbonothioylthio)­pentanoic acid), and one synthesized by conventional free radical polymerization, in the absence of any RAFT agent. Each polymer was then subjected to flash nanoprecipitation in aqueous solution, and the resulting suspensions were analyzed by DLS (Figure S3) to evaluate their ability to form nanostructures. The outcomes clearly highlight the critical role of CTA hydrophobicity in driving the nanoparticle formation. The PMAA synthesized using the dodecyl-containing CTA yielded well-defined nanoparticles with an average hydrodynamic diameter around 110 nm and a narrow size distribution. In contrast, the formulation obtained with the phenyl-containing CTA produced significantly larger and more polydisperse structures, with an average size close to 260 nm and high PDI. Finally, the polymer obtained by free radical polymerization exhibited poor self-assembly ability, forming irregular aggregates with an average diameter exceeding 700 nm and a very broad distribution. These results confirm that the incorporation of a long aliphatic chain through the RAFT agent is essential to induce self-assembly into uniform nanostructures. Although the phenyl group in the second CTA provides some degree of hydrophobicity, it is clearly insufficient when compared to the stronger hydrophobic driving force imparted by a C_12_ alkyl chain. The results obtained by FRP also suggest that PMAA alone is water-soluble and does not self-assemble under the same conditions, supporting the conclusion that the hydrophobic C_12_ chains form the actual core of the nanoparticles. Furthermore, this core–shell model is consistent with the temperature dependent behavior observed via DLS. Upon heating, the thermoresponsive EG_2_MA-*co*-NIPAM block collapses, leading to a decrease in hydrodynamic diameter and a more compact core.

The PMAA block, which remains solvated and partially ionized at neutral pH, provides steric and electrostatic stabilization, preventing aggregation despite the collapse of the thermoresponsive corona.

### Nanoparticle Synthesis and Characterization

The amphiphilic block copolymers described in Table 2 were employed to obtain dual-responsive NPs through a self-assembly process in aqueous media. Although spontaneous self-assembly can lead to micellar structures, the control over particle size and dispersity is often limited, resulting in heterogeneous suspensions with broad distributions. To overcome these limitations and ensure reproducible and efficient nanoparticle formation, a flash nanoprecipitation approach was adopted.^50–52^ Nanoparticles were prepared by rapidly injecting a polymer solution into water under controlled mixing conditions, leading to the formation of stable colloidal systems. The resulting NPs physicochemical properties are reported in Table 3 together with their hydrodynamic diameters and polydispersity indexes (PDI) as determined by DLS (Figure S4). For all four polymer formulations, the process yielded monodisperse nanoparticles with size below 182 nm and PDI values below 0.11, confirming the effectiveness of the strategy and the suitability of the copolymer architecture for colloidal stabilization. Although particles in this size range are not expected to be directly cleared by renal filtration, their polymeric composition ensures progressive degradation and elimination through physiological pathways, preventing undesirable long-term retention.^53–55^

**Table 3:** Physicochemical Proprieties and Responsive Behavior of Nanoparticles, Including: Hydrodynamic Diameter at Room Temperature (*D*_0_), Polydispersity Index, pH Responsivity, Diameter Variation Above the LCST (Δ*D*_h_), Cloud Point Determined by UV–Vis and Critical Micelle Concentration

#	sample	*D*_0_ (nm)	PDI	pH respectively	CP-DLS (°C)	Δ*D* (nm)	CP-Uv (°C)	CMC (mg/L)
A	(PMAA)_50_-*b*-(EG_2_MA_17_-*co*-NIPAM_33_)	160.3	0.061	6	37	88.17	39	3.43
B	(PMAA)_50_-*b*-(EG_2_MA_25_-*co*-NIPAM_25_)	125.6	0.080	6	32	58.16	33	3.45
C	(PMAA)_50_-*b*-(EG_2_MA_46_-*co*-NIPAM_4_)	121.2	0.106	6	30	78.17	31	2.54
D	(PMAA)_50_-*b*-(EG_2_MA_50_-*co*-NIPAM_50_)	181.3	0.083	6	40	63.2	41.5	1.63

The particle size was found to correlate with the relative length of the two blocks within the copolymer. In particular, a trend was observed where an optimal hydrophilic/hydrophobic balance minimized the final NP size, in agreement with previous observations in literature.^56^ Copolymers with a shorter hydrophilic segment formed more compact and smaller nanoparticles, while those with a longer hydrophilic fraction exhibited increased swelling behavior, leading to slightly larger dimensions. This behavior can be attributed to the higher hydration degree of the more hydrophilic compositions, which tend to form looser assemblies in aqueous environments. The smallest particle size was obtained for the copolymer formulation in which the molecular weights of the hydrophilic and hydrophobic blocks were comparable. The morphology of the nanoparticles was confirmed by TEM, which revealed spherical and uniform micellar structures in agreement with DLS data (Figure 4).

**Figure 4:**
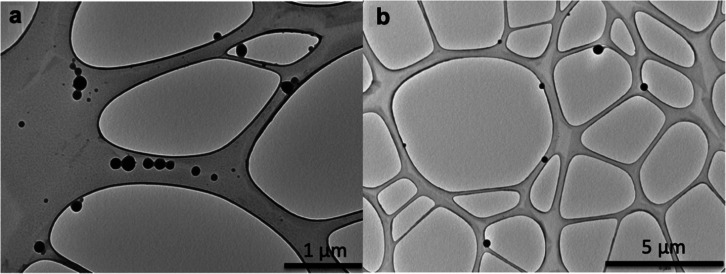
TEM image of sample D (a) and sample A (b) nanoparticle suspension on grid.

The critical micelle concentration (CMC) of each copolymer was also determined using pyrene as a hydrophobic fluorescent probe. At low polymer concentrations, *I*_3_/*I*_1_ values between 0.57 and 0.55 indicated pyrene dispersion in the aqueous phase and the absence of micelle formation. As the polymer concentration increased, a gradual increase in the *I*_3_/*I*_1_ ratio was observed, indicating the formation of hydrophobic domains and micelle nucleation (Figure S5). The CMC was determined as the inflection point in the *I*_3_/*I*_1_ vs concentration plot and was found to range between 1.63 and 3.45 mg/L, consistent with literature values reported for similar amphiphilic block copolymers.^57^ Interestingly, a decreasing trend in CMC was observed as the overall hydrophilicity of the thermoresponsive block decreased, suggesting that a more hydrophobic copolymer composition promotes micelle formation at lower concentrations, likely due to enhanced core–shell segregation in aqueous media. The thermoresponsive behavior of the synthesized nanoparticles was systematically investigated through both DLS and UV–vis turbidimetry. The four amphiphilic block copolymers studied differed either in the ratio between the pH-responsive and thermoresponsive blocks, or in the composition of the thermoresponsive segment itself, specifically in the relative content of EG_2_MA and NIPAM units (Table 1).

DLS measurements were employed to monitor the evolution of nanoparticle size as a function of temperature (Table 3). All samples showed a sigmoidal decrease in hydrodynamic diameter upon heating above the LCST, which was associated with the coil-to-globule transition of the thermoresponsive outer shell (Figure 5a,b). This transition is driven by the dehydration and collapse of the P­(EG_2_MA-*co*-NIPAM) block, which shifts from a hydrated, expanded coil to a collapsed, hydrophobic globule. Therefore, the nanoparticles shrink in size rather than swell, a behavior typical of polymeric systems bearing a shell that dominates the overall hydrodynamic profile. As expected, the magnitude of this size reduction (Δ*D*) varied across the different formulations, reflecting differences in the hydrophilic/hydrophobic balance and in chain mobility. Among the tested formulations, sample A (EG_2_MA/NIPAM = 17/33) exhibited a cloud point of 37 °C and a pronounced diameter decrease, from an initial size of 160.3 nm to over 70 nm (Δ*D* = 88.17 nm). This large contraction is indicative of a highly mobile and responsive corona, favored by a relatively low EG_2_MA content and a high NIPAM fraction. Conversely, sample B, with an equimolar EG_2_MA/NIPAM ratio (25/25), showed a cloud point of 32 °C and a reduced diameter change (Δ*D* = 58.16 nm), indicating that compositional symmetry attenuates the responsiveness. Sample C, which contained a high EG2MA fraction (46/4), displayed the lowest cloud point (30 °C), while still exhibiting significant swelling (Δ*D* = 78.17 nm), likely due to the enhanced hydration of the shell at lower temperatures. Interestingly, sample D, featuring a 1:2 ratio between the pH-responsive block (PMAA) and the thermoresponsive segment (P­(EG_2_MA-*co*-NIPAM), with an internal 50/50 composition), exhibited the highest LCST (40 °C) and the most pronounced size reduction (Δ*D* = 100 nm). This behavior suggests that increasing the overall content of the thermoresponsive block enhances the hydrophilicity of the nanoparticle shell, resulting in a delayed LCST transition. However, once the transition is triggered, the dehydration of the hydrophilic chains is more dramatic, leading to a sharper coil-to-globule collapse and a greater decrease in hydrodynamic diameter.

**Figure 5:**
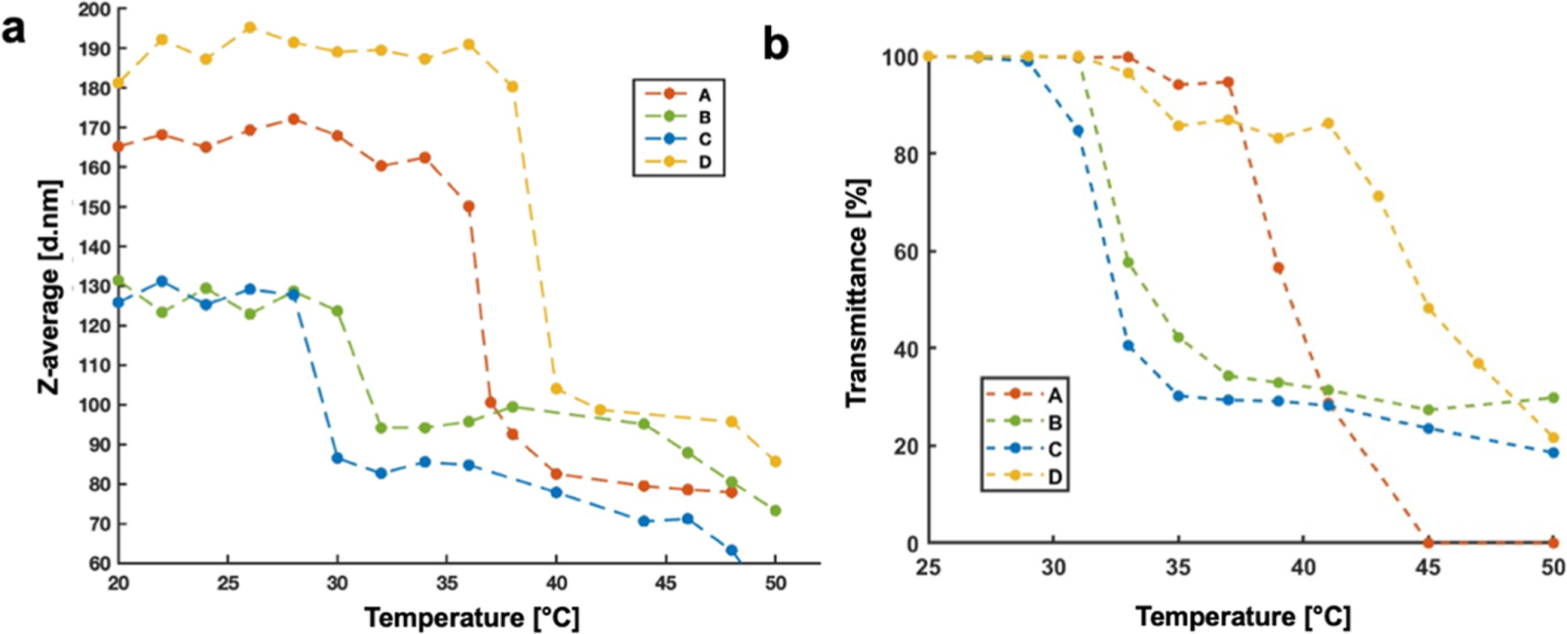
(a) Temperature-dependent variation of the hydrodynamic diameter (*Z*-average) of nanoparticles measured by DLS for samples A–D, showing the coil-to-globule transition associated with the LCST behavior. (b) Corresponding temperature-dependent optical transmittance at 500 nm for nanoparticle suspensions recorded by UV–vis spectroscopy, confirming the phase transition observed by DLS; for sample A (red) sample B (green) sample C (blue) sample D (yellow).

These DLS results were complemented by UV–vis turbidimetry, which was employed to determine the cloud point temperature (T_cp) associated with the demixing behavior of the polymer solution in PBS (Table 3). The transmittance of the NP suspensions was recorded while gradually increasing the temperature from 25 to 50 °C. For all samples, a clear decrease in transmittance was observed upon approaching the LCST, reflecting the formation of hydrophobic domains and increased light scattering. The cloud point, taken as the temperature at which the transmittance dropped by 50%, showed good agreement with the CP values derived from DLS, ranging from 31 to 42 °C across the series.

Importantly, the phase transition remained sharp and reversible for all samples, as confirmed by DLS and UV–vis analyses performed first during heating and then on the same sample during cooling. The observed correlation between block composition, LCST, and nanoparticle swelling behavior underscores the possibility to finely tune the thermal responsiveness of the system through controlled copolymer design.

These features make the resulting nanoparticles promising candidates for biomedical applications, especially in drug delivery scenarios requiring responsiveness in the range 30–42 °C, such as in the vaginal environment or in inflamed tissues. PMAA can be classified as a weak polyacid, whose carboxyl groups (−COOH) undergo pH-dependent ionization. This property makes PMAA the main driver of the pH-responsiveness of the NPs reported in this work. At acidic pH, the carboxyl groups are predominantly protonated, reducing electrostatic repulsion and increasing interchain hydrogen bonding, which promotes inter- and intramolecular interactions. As a result, NPs tend to aggregate, leading to an increase in hydrodynamic size. In contrast, at alkaline pH values, the carboxyl groups become deprotonated and negatively charged, generating strong electrostatic repulsion among polymer chains. This destabilizes the NP structure and may lead to disassembly or disruption of the nanostructures.^38^ These theoretical assumptions were supported by both DLS and UV–vis spectroscopy measurements performed at different pH values. As shown in Figure 6a, DLS analysis revealed a marked increase in the average particle size starting from pH values between 6 and 5, confirming the onset of aggregation driven by the protonation of the PMAA block. At basic pH, the DLS signal remained centered around a relatively constant average size, but with a progressive increase in PDI, which may indicate the progressive hydrolysis of the polymer structure. The pH-dependent behavior was also confirmed by UV–vis turbidimetry measurements (Figure 6b). The transmittance curve shows a characteristic sigmoidal trend, with low transmittance at acidic pH, due to light scattering by aggregates, and a steep increase at higher pH values, where particle destabilization results in reduced scattering. This apparent shift toward a higher pH threshold can be explained by microenvironmental confinement effects within the nanoparticle core. In such a restricted and partially hydrophobic environment, the local dielectric constant is reduced and the PMAA chains experience cooperative protonation and interchain hydrogen bonding, which stabilize the protonated state and effectively increase the apparent p*K*_a_.^23^ Finally, zeta potential measurements (Figure 6c) further confirmed the surface charge modulation.

**Figure 6:**
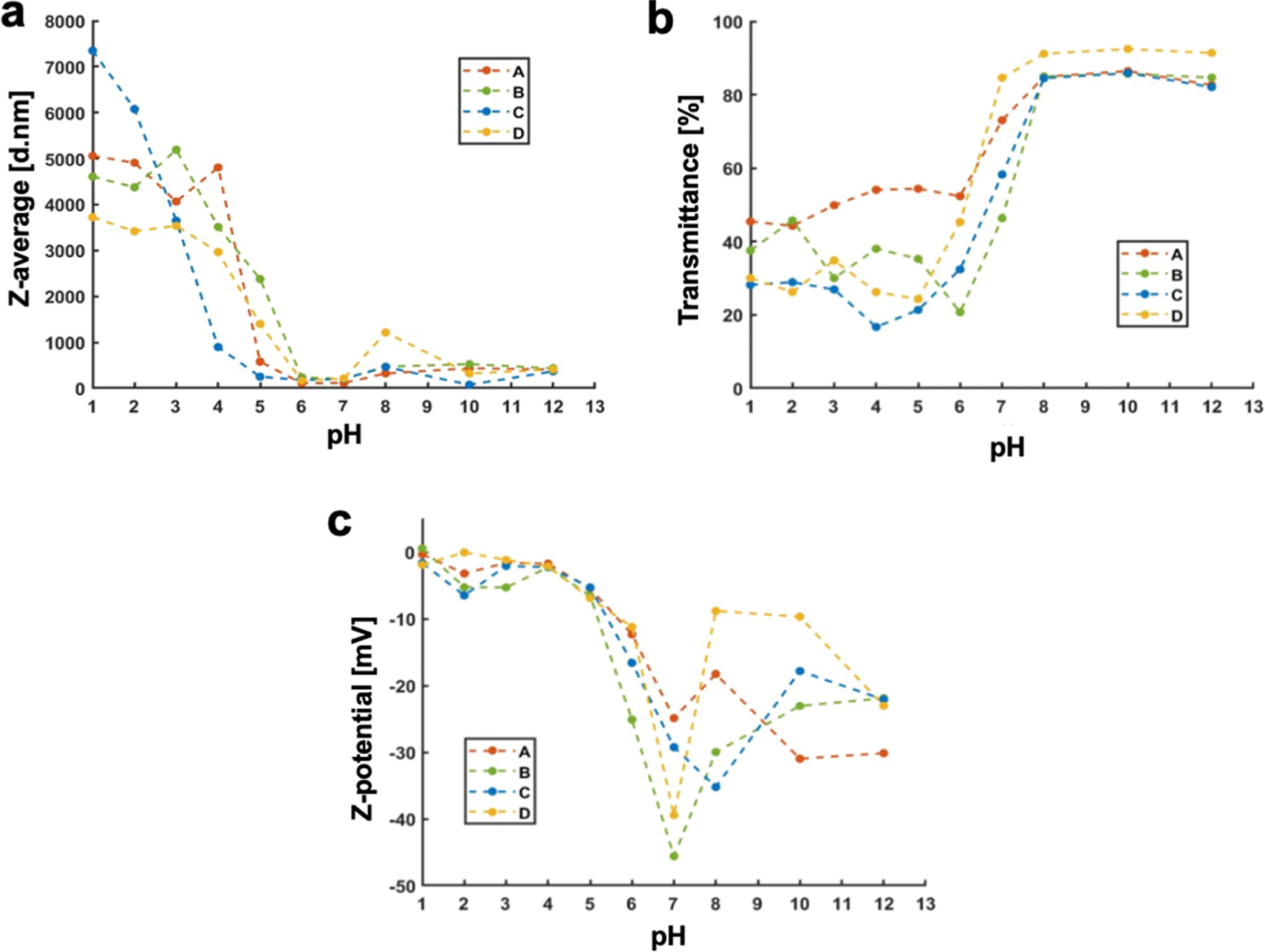
(a) Variation of the hydrodynamic diameter (*Z*-average) measured by DLS as a function of pH, showing pronounced aggregation under acidic conditions (pH < 6) due to protonation of the PMAA block and hydrogen-bond–driven interparticle association. (b) Corresponding optical transmittance at 500 nm recorded by UV–vis spectroscopy, confirming the increased turbidity at low pH caused by nanoparticle aggregation and the recovery of transparency at higher pH values where deprotonation restores colloidal stability; (c) zeta potential profiles of the nanoparticles as a function of pH, evidencing the surface charge reversal from nearly neutral values under acidic conditions to highly negative potentials at basic pH, consistent with the ionization of carboxylic groups; for sample A (red) sample B (green) sample C (blue) sample D (yellow).

At acidic pH, the zeta potential approaches neutrality, reflecting the protonation of the carboxylic groups. As the pH increases, the zeta potential becomes increasingly negative, consistent with the progressive deprotonation of PMAA and enhanced colloidal instability. These results collectively demonstrate that the synthesized NPs are effectively pH-responsive, with their structural integrity and colloidal behavior being strongly modulated by the protonation state of PMAA chains, particularly in the pH region below 6.

### Drug Release

To evaluate the responsiveness of the NPs to external stimuli, drug release experiments were performed by encapsulating two model compounds: fluorescein isothiocyanate (FITC), a poorly soluble and moderately hydrophobic molecule, and 5-fluorouracil (5-FU), a hydrophilic and highly soluble anticancer drug.^58^ Both compounds were loaded using a flash nanoprecipitation protocol, which allowed for the rapid formation of polymeric NPs under kinetically controlled conditions. The coexistence of both FITC and 5-FU within the same nanoparticle formulation can be explained by the core–shell organization of the micelles. The hydrophobic FITC preferentially partitions into the compact inner core formed by the dodecyl chain of the RAFT agent, while the hydrophilic 5-FU is mainly retained at the core–shell interface or within the hydrated outer corona, where hydrogen bonding with PMAA carboxylic groups promotes its stabilization.^38^ The encapsulation of FITC was quantified via UV–vis spectroscopy at its maximum absorption wavelength (λ = 495 nm in PBS), while 5-FU, due to its relatively low molar absorptivity at 265 nm, was quantified using high-performance liquid chromatography (HPLC). Encapsulation efficiency (EE %) and drug loading (DL %) are reported in Table S1 for the different polymeric formulations. Both encapsulation efficiency and drug loading values were found to be satisfactory across all tested formulations. FITC consistently displayed slightly higher loading and encapsulation values compared to 5-FU, which is rationalized by its greater affinity for the hydrophobic micellar core, favoring strong noncovalent interactions with the polymer chains. In contrast, the hydrophilic nature of 5-FU tends to promote localization in the outer corona of the NPs, closer to the aqueous interface. This trend is inverted in sample D, where the encapsulation efficiency of 5-FU reaches its highest value (91.02%), while that of FITC slightly decreases (82.50%).

This inversion suggests that the extended hydrophobic segment in formulation D may enhance the retention of more hydrophilic drugs within the nanostructure, possibly by altering the internal packing or increasing polymer/drug interactions near the core/corona interface. To examine the effect of temperature on drug release, parallel dialysis experiments were carried out at two temperatures: one below and one above the lower critical solution temperature (LCST) of the polymer. Cumulative release profiles of both drugs from NPs are shown in Figure 7a–d.

**Figure 7:**
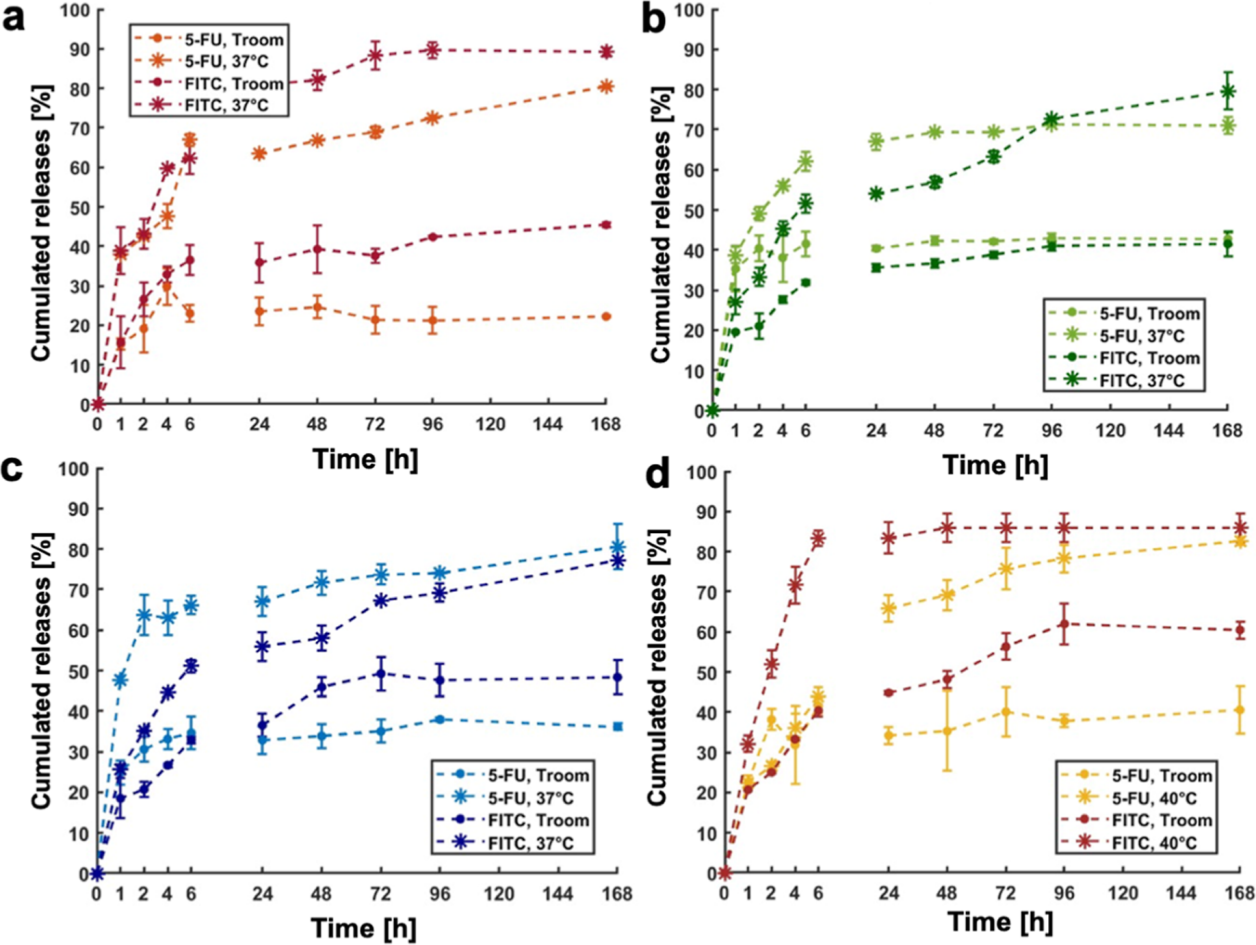
Cumulated release from NPs vs time (hours) of 5-FU (light color), FITC (dark color) at RT (full points) and 37 °C (asterisk) for sample A (red) (a), sample B (green) (b), sample C (blue) (c), sample D (yellow) (d).

All samples exhibited an initial rapid release phase during the first hours, consistent with Fick’s first law of diffusion.

This burst release is typically attributed to drug molecules located near or on the nanoparticle surface, where they are less effectively retained and more readily diffuse into the surrounding aqueous medium due to the high concentration gradient.^38^ This initial burst was markedly more pronounced at temperatures above the LCST, further confirming the temperature-sensitive behavior of the NPs. Above the LCST, polymer collapse leads to the disruption of the core–shell structure, reducing the diffusive barrier and facilitating drug release. Beyond the early diffusion-driven release, sustained release was observed in the longer term, indicating the interplay of additional mechanisms such as polymer matrix relaxation, erosion, and reorganization. Importantly, convective effects were excluded from the experimental design by maintaining static conditions during the entire release period. The differences in release profiles between FITC and 5-FU further underline the impact of molecular hydrophobicity. FITC, being more hydrophobic, is retained longer within the core and released more gradually. Conversely, the hydrophilic 5-FU is preferentially distributed in the outer shell region of the NP, from which it is more readily released into the surrounding medium. The correlation between the apparent LCST of each formulation and its release kinetics is illustrated in Figure S6. Overall, these results demonstrate the effective encapsulation and stimuli-responsive release behavior of the developed polymeric nanoparticles, highlighting their potential utility as drug carriers capable of modulating release profiles in response to environmental temperature changes. To validate the qualitative observations described above, the release kinetics were further analyzed using the Korsmeyer–Peppas model.^42^ The logarithm of cumulative release versus the logarithm of time (Figure 8) yielded linear correlations with coefficients of determination (*R* ^2^) above 0.94, confirming the validity of the model. The calculated *n* values ranged between 0.26 and 0.40, indicating a quasi-Fickian diffusion mechanism characteristic of drug transport from a nonswellable polymeric matrix. This behavior suggests that diffusion through the nanoparticle shell is the rate-limiting step, consistent with the relatively compact architecture of the core–shell micelles.

**Figure 8:**
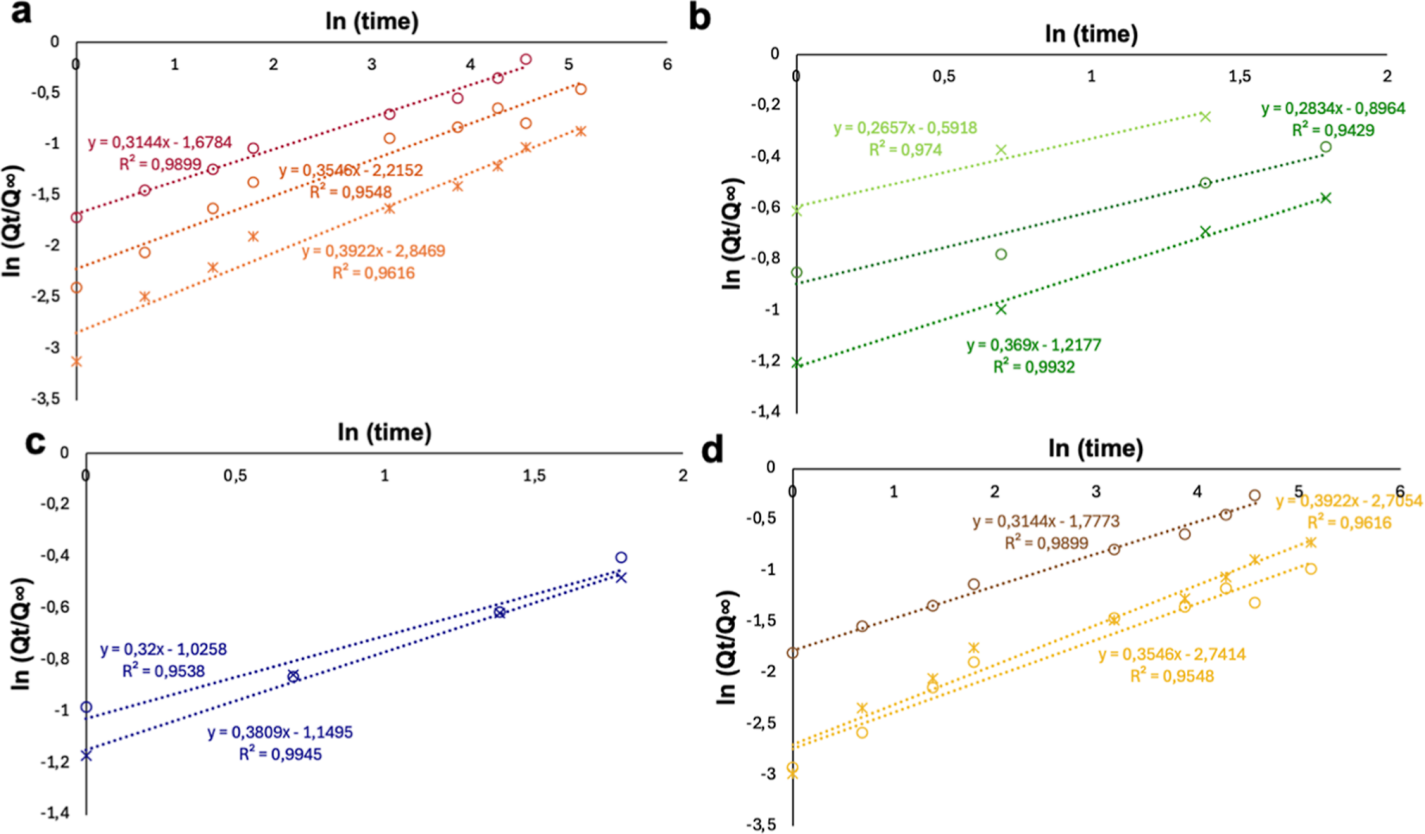
Logarithmic plots of cumulative fractional release (ln Q_t_/*Q*_∞_ vs ln*t*) for 5-FU (light color), FITC (dark color) at RT (full points) and 37 °C (cross) for sample A (red) (a), sample B (green) (b), sample C (blue) (c), sample D (yellow) (d). Linear fits were used to determine the diffusion exponent (n) and kinetic constant (k) according to the Korsmeyer–Peppas equation *Q*_t_/*Q*_∞_ = *k*·*t* ^*n*^.

Slight variations in the diffusion exponent among formulations can be attributed to differences in nanoparticle geometry and polydispersity, which modulate the diffusional path length and matrix relaxation. An *n* value of 0.432 ± 0.007 is in close agreement with classical Fickian diffusion from spherical polymeric systems, as previously described for PNIPAM- and PMAA-based micellar carriers.^59^

To further assess the potential of the dual-stimuli design, the formulation exhibiting the most physiologically relevant A-LCST (37 °C) was selected and tested using pyrene as a model strongly hydrophobic molecule. Pyrene was chosen because of its low aqueous solubility and strong affinity for hydrophobic polymer domains, which makes it a stringent probe for evaluating the efficiency of thermo- and pH-triggered release mechanisms.

Indeed, previous studies have reported that thermoresponsive systems based solely on NIPAM often show limited release of hydrophobic drugs when exposed only to thermal stimuli, due to strong interactions between the collapsed polymer core and the cargo.^29^ As shown in Figure 9, the cumulative release of pyrene markedly increased under combined acidic and thermal conditions (37 °C, pH 5.5), confirming the cooperative role of the two stimuli.

**Figure 9:**
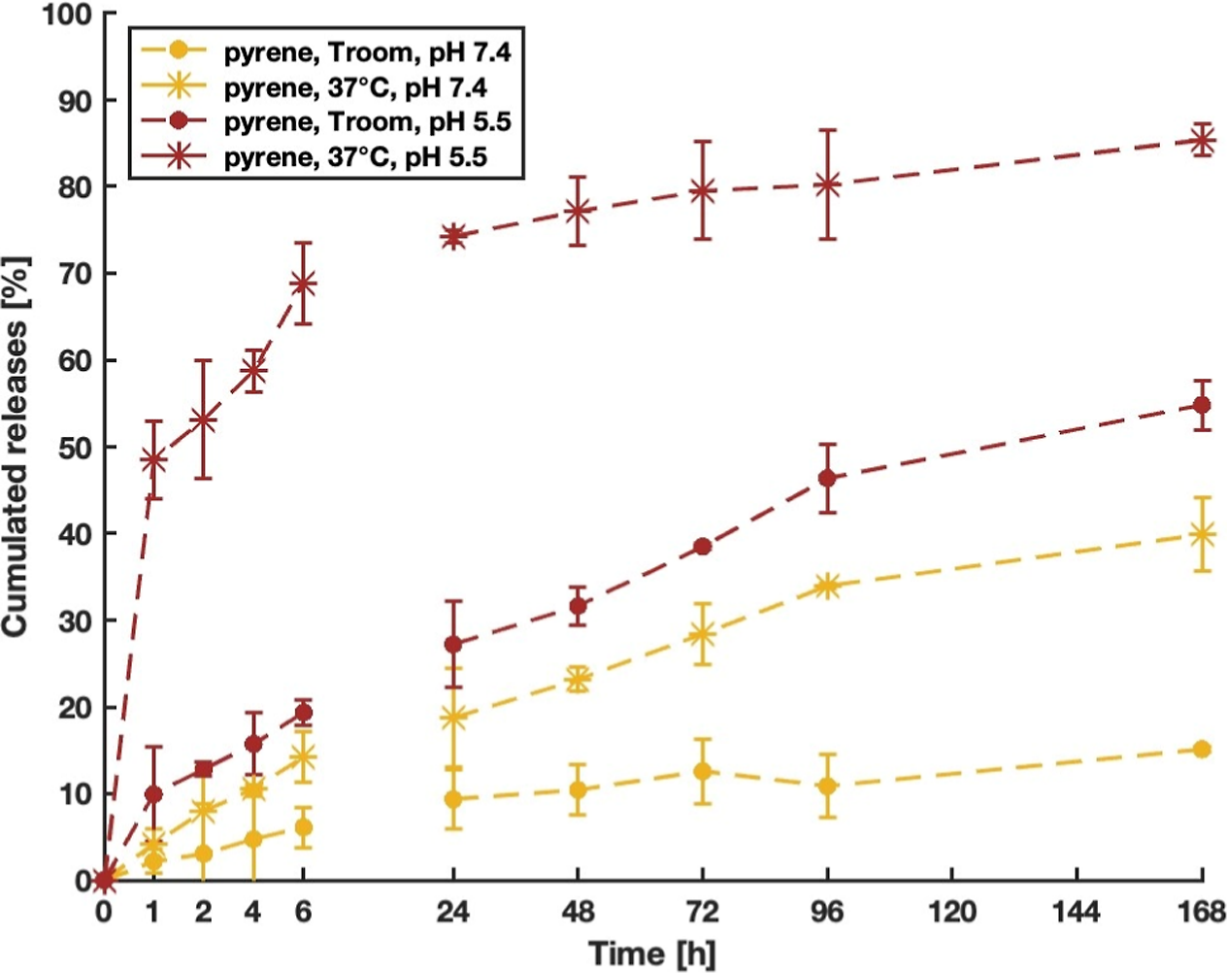
Cumulated release from NPs vs time (hours) of pyrene at RT and pH 7.4 (full points, yellow); 37 °C and pH 7.4 (asterisk, yellow); RT and pH 5.5 (full points, red); 37 °C and pH 5.5 (asterisk, red) for sample A.

The protonation of methacrylic acid groups at acidic pH reduces electrostatic repulsion and increases matrix hydrophobicity, while the LCST transition promotes a hydrophilic-to-hydrophobic collapse that enhances molecular mobility and drives the expulsion of the hydrophobic probe.^60^

The result is a synergistic and accelerated release, with nearly complete diffusion under combined conditions, compared to the limited release observed under single-stimulus environments (either pH 5.5 or 37 °C alone).

### In Vitro Studies

To enable the tracking of cellular uptake and intracellular localization, rhodamine B was covalently incorporated into the PMAA backbone to generate fluorescently labeled nanoparticles. DLS analysis confirmed the formation of monodisperse nanostructures with hydrodynamic diameters consistent with those of the nonfluorescent formulation. No secondary populations or significant changes in polydispersity index were observed, corroborating the structural stability of the labeled nanoparticles. A crucial prerequisite for their biological application is the verification that the fluorophore remains stably confined within the nanostructure. Dialysis experiments revealed no detectable rhodamine signal in the external medium over a one-week monitoring period, confirming excellent retention stability and excluding dye leakage. Based on their thermoresponsive behavior, two formulations (sample A, LCST = 37 °C; sample D, LCST = 40 °C) were selected for biological testing. Both exhibited excellent biocompatibility in OVCA433 and HeLa cells, as assessed by MTT assay. Before performing the biological assays, the stability and thermoresponsiveness of all four nanoparticle formulations were evaluated in the same culture medium used for cell experiments (DMEM supplemented with 10% FBS). The nanoparticles maintained colloidal stability and exhibited the same LCST-dependent behavior observed in PBS, confirming that the dual-responsive properties are preserved under physiologically relevant conditions. Cell viability remained close to 100% at low concentrations, with only a slight decrease observed at the highest tested dose (2.5 mg/mL). These findings confirm the noncytotoxic profile of the nanocarriers and support their suitability for biomedical applications (Figure S7).

The uptake of fluorescent NPs was evaluated by both flow cytometry (FC) and confocal fluorescence microscopy. FC analyses were conducted on OVCA433 and HeLa cells after 2 h, 6 and 24 h of incubation with rhodamine-labeled NPs. As reported in Figure 10a, a significant shift in fluorescence distribution was already evident after 2 h compared to the control group, suggesting that nanoparticle internalization occurs rapidly and efficiently. The mean fluorescence intensity (MFI) remained stable or slightly increased over time, but the full separation of fluorescence peaks observed as early as 2 h indicates that cellular uptake was largely completed within the first incubation period. The nanoparticles were not ligand-functionalized and the encapsulated cargos (5-FU, FITC) do not act as targeting moieties. Given their hydrodynamic diameter (120–180 nm) and hydrated PNIPAM/PEG corona, the nanoparticles are expected to enter cells primarily through energy-dependent endocytosis, involving clathrin-mediated and macropinocytic routes, as typically reported for polymeric micelles of similar size.^61^

**Figure 10:**
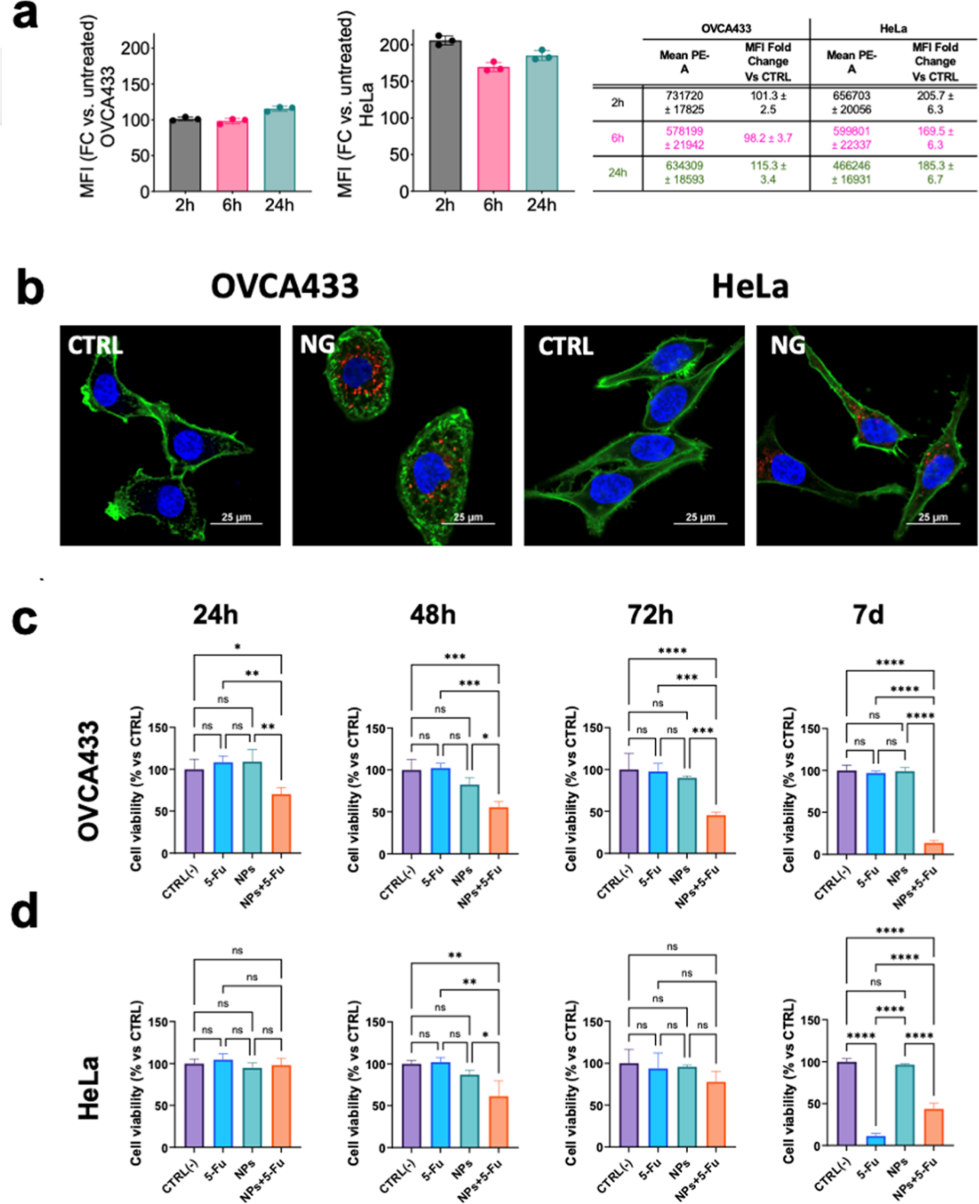
(a) Mean fluorescence intensity of untreated cells (red) and cells treated with fluorescent NPs (green) at 2 h, 6 h and 24 h after incubation; (b) representative confocal micrographs of NPs (in red) internalization in the OVCA433 and HeLa cells before and after 4 h of incubation; MTT assay on OVCA433 (c) and HeLa (d) cells untreated (purple), 5-FU solution (blue), treated with pristine NPs (green), and 5-FU-loaded NPs (orange) at 24 h, 48 h and 72 h.

These observations were further validated by confocal microscopy. Figure 10b shows representative images of OVCA433 and HeLa cells, respectively, following treatment with fluorescent NPs. Red fluorescence signals confirmed the presence of nanoparticles inside the cells. Nuclei and plasma membranes were counterstained with blue (DAPI) and green (WGA-FITC) dyes, respectively.^62^ To evaluate the performance of the nanomaterials as anticancer drug nanocarriers, HeLa and OVCA433 cells were incubated with 5-FU-loaded NPs. A deliberately low drug concentration was selected, insufficient to induce acute cytotoxic effects when administered in its free form.^63,64^ Cell viability was monitored over 24 h, 48 h, 72 h, and 7 days. As evidenced in Figure 8d and 10c, treatment with 5-FU-loaded NPs resulted in a marked reduction in cell viability compared to the group exposed to the free drug at the same nominal concentration. This effect was particularly evident in OVCA433 cells, which are typically characterized by intrinsic resistance to 5-FU. In ovarian cancer, 5-FU efficacy is severely limited by thymidylate synthase (TS) overexpression, which hampers nucleotide synthesis and reduces drug sensitivity. The enhanced cytotoxicity observed in the OVCA433 model suggests that nanoparticle-mediated delivery may help bypass these barriers by promoting local accumulation and sustained release, in agreement with recent findings highlighting TS-related resistance pathways.^65^ Future developments will focus on encapsulating clinically relevant anticancer agents such as cisplatin, paclitaxel, and doxorubicin to confirm the versatility and translational relevance of the nanoplatform. In contrast, HeLa cells are inherently much more responsive to 5-FU, and this high susceptibility was reflected in data showed in Figure 10d: after 7 days, the free drug outperformed the nanoparticle formulation, likely due to cumulative drug exposure in the absence of medium renewal.^66^ The divergent pharmacological profiles observed in OVCA433 and HeLa cells can be rationalized on the basis of known mechanisms of 5-FU resistance. In ovarian cancer, including OVCA433, 5-FU shows limited efficacy, which has been mechanistically associated with increased expression of thymidylate synthase (TS), the main target enzyme of 5-FU.^67^

Elevated TS levels allow cells to maintain nucleotide biosynthesis despite drug exposure, thereby conferring a resistant phenotype.^68^ In addition, efflux pumps of the ATP-binding cassette (ABC) transporter family, most notably ABCC5/MRP5 and ABCC4/MRP4, can actively export phosphorylated 5-FU metabolites, reducing their intracellular retention and further diminishing cytotoxicity.^69^ Nanoparticle-mediated delivery may mitigate both mechanisms by facilitating intracellular accumulation of the drug and modulating its subcellular distribution, partially overcoming the efflux barrier and saturating TS-mediated resistance. This explains why OVCA433, generally unresponsive to 5-FU, exhibited enhanced susceptibility when exposed to the NP formulation. Conversely, HeLa cells are known to be intrinsically sensitive to 5-FU, with reported IC_50_ values in the low micromolar range, and thus the incremental benefit of NP-mediated delivery is less pronounced.^70^ Therefore, higher doses are typically required to achieve therapeutic efficacy, increasing the risk of off-target toxicity and systemic side effects. Moreover, the freely diffusing drug lacks the ability to concentrate and retain locally within the tumor microenvironment, limiting its therapeutic window. Encapsulation within pH- and thermoresponsive nanocarriers not only enhances site-specific delivery, but also enables sustained and localized release, further amplifying the cytotoxic effect even at reduced concentrations. These preliminary in vitro results demonstrate that the designed nanoparticle systems can selectively enhance drug efficacy in tumor cells via intracellular delivery, even at very low drug concentrations. Future work will extend this validation to more physiologically relevant models, including 3D tumor spheroids and organ-on-chip systems, to simulate complex tissue microenvironments and assess the therapeutic performance under dynamic flow conditions before progressing to in vivo studies.

## Conclusions

This study demonstrates the successful design of dual pH- and thermoresponsive nanoparticles for precision drug delivery in gynecological cancers. The block copolymers P­(MAA)-*b*-P­(EG_2_MA-*co*-NIPAM), synthesized via RAFT polymerization, enabled fine modulation of the LCST within the physiological range and allowed for highly reproducible self-assembly into monodisperse nanoscale carriers. The use of a long-chain hydrophobic CTA played a pivotal role in core stabilization and encapsulation efficiency, supporting drug loading for both hydrophilic and hydrophobic molecules. The nanoparticles exhibited well-defined phase transitions in response to both pH and temperature variations, reflecting their suitability for releasing therapeutics in the acidic and inflamed tumor microenvironment. Drug release experiments demonstrated controlled and stimuli-responsive release profiles, while in vitro studies confirmed excellent cytocompatibility of the pristine nanocarriers and rapid cellular uptake. Notably, 5-FU-loaded NPs significantly outperformed the free drug in reducing the viability of tumoral OVCA433 cell line, underlining the benefits of intracellular delivery and sustained release. These results validate the dual-responsive platform as a highly promising candidate for site-specific and effective drug delivery in oncological applications. The rational design approach adopted here lays the foundation for next-generation smart nanomedicines capable of adapting to the complexity of the tumor microenvironment.

## Supplementary Material



## Notes

^1^H NMR spectra of the synthesized copolymers during RAFT polymerization; GPC chromatograms of the synthesized copolymers; DLS analyses of nanoparticles; critical micelle concentration determination; encapsulation efficiency and drug loading data for FITC and 5-FU; correlation between apparent LCST and release kinetics; and in vitro biocompatibility assays on representative formulations (PDF)
