# Supplementary material for: pH-Thermo Dual-Responsive Polymeric Nanoparticles for Women’s Health: Dual Action Against Cervical and Ovarian Cancer Cells
Source: ACS Appl Mater Interfaces. 2025 Oct 28;17(45):61888–904. doi: 10.1021/acsami.5c18234 (PMC12616599; doi:10.1021/acsami.5c18234)
Supplement: Supplementary file 1 [file am5c18234_si_001.pdf]

Supporting Information

pH-Thermo Dual-Responsive Polymeric Nanoparticles for Women's Health: Dual Action against Cervical and Ovarian Cancer Cells

Giuseppe Nunziata [1], Emanuele Limiti [2,3], Dania Aramini [1], Marco Nava [1], Luca Moretti [3,4], Alberto Rainer [3,5], Mattia Sponchioni [1] and Filippo Rossi [1,]*

[1] Department of Chemistry, Materials and Chemical Engineering "Giulio Natta", Politecnico di Milano, piazza Leonardo da Vinci 32, 20133, Milan, Italy

[2] Department of Science and Technology for Sustainable Development and One Health, Università Campus Bio-Medico di Roma, via Álvaro del Portillo 21, 00128 Rome, Italy

[3] CNR NANOTEC, Istituto di Nanotecnologia, Via Monteroni, 73100 Lecce, Italy

[4] Department of Science and Engineering of Matter, Environment and Urban Planning, Università Politecnica delle Marche, via Brecce Bianche 12, 60131 Ancona, Italy

[5] Department of Engineering, Università Campus Bio-Medico di Roma, via Álvaro del Portillo 21, 00128 Rome, Italy

E-mail: filippo.rossi@polimi.it

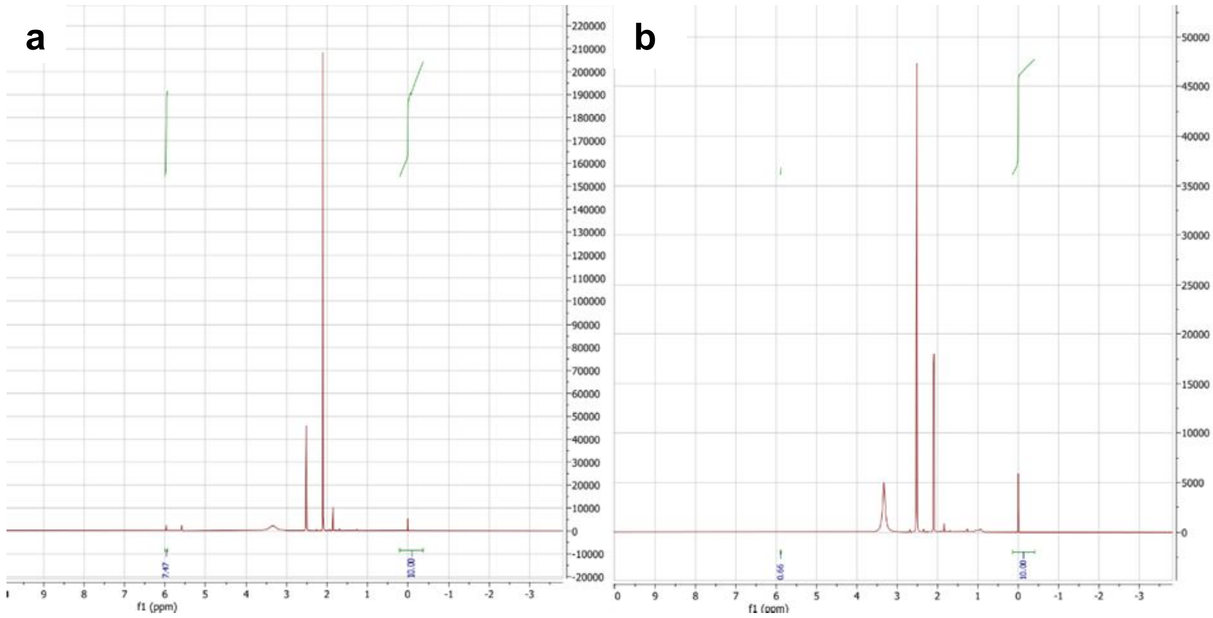

Figure S1. ^{1}H-NMR in dmso-d_6 of PMAA during RAFT polymerization of MAA monomers at 0h (a) and 24h (b). MAA monomer peak at 6.1 ppm decreased relative to the TSP signal at 0 ppm.

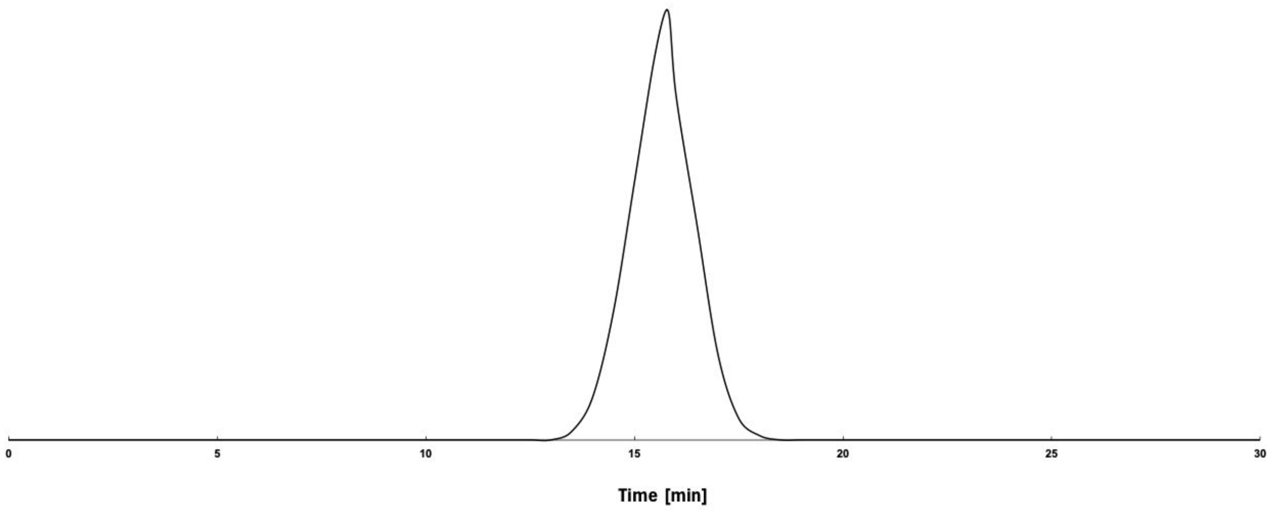

Figure S2. GPC chromatograms with relative retention time (dotted line) of (PMAA)-b-P(EG$_2$MA-co-NIPAM) with an unimodal and narrow molecular weight distribution.

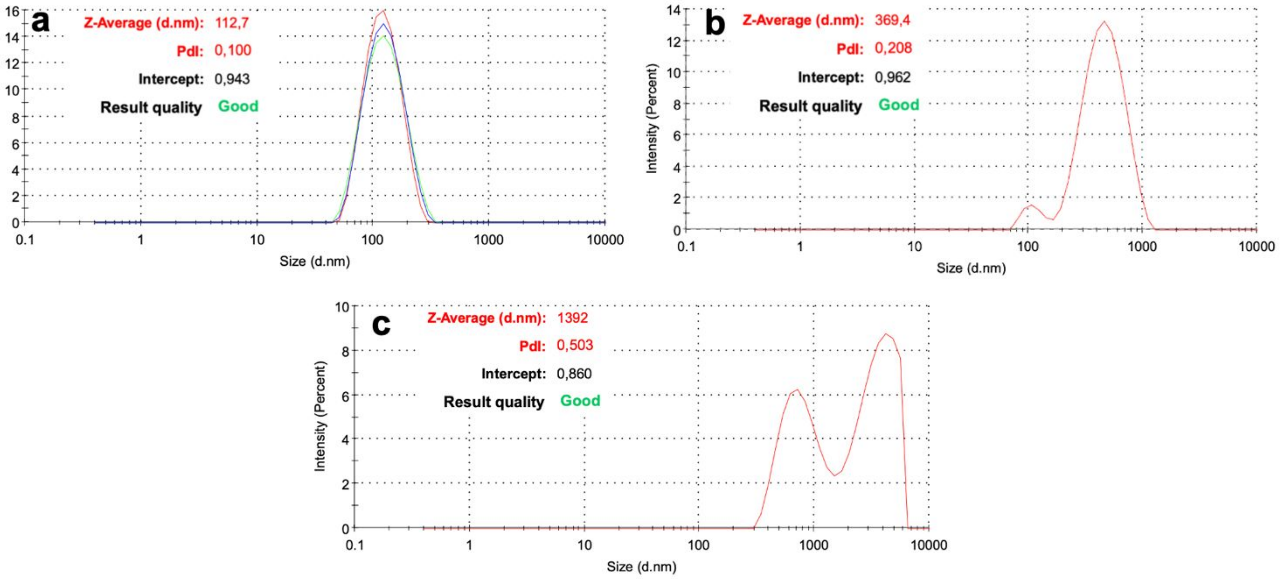

Figure S3. DLS PMAA-based nanoparticles synthesized: **(a)** using a highly hydrophobic CTA containing a dodecyl chain (4-Cyano-4-[(dodecylsulfanylthiocarbonyl)sulfanyl]pentanoic acid); **(b)** employing a less hydrophobic CTA bearing a phenyl group (4-cyano-4-(phenylcarbonothioylthio)pentanoic acid); **(c)** synthesized by conventional free radical polymerization, in the absence of any RAFT agent.

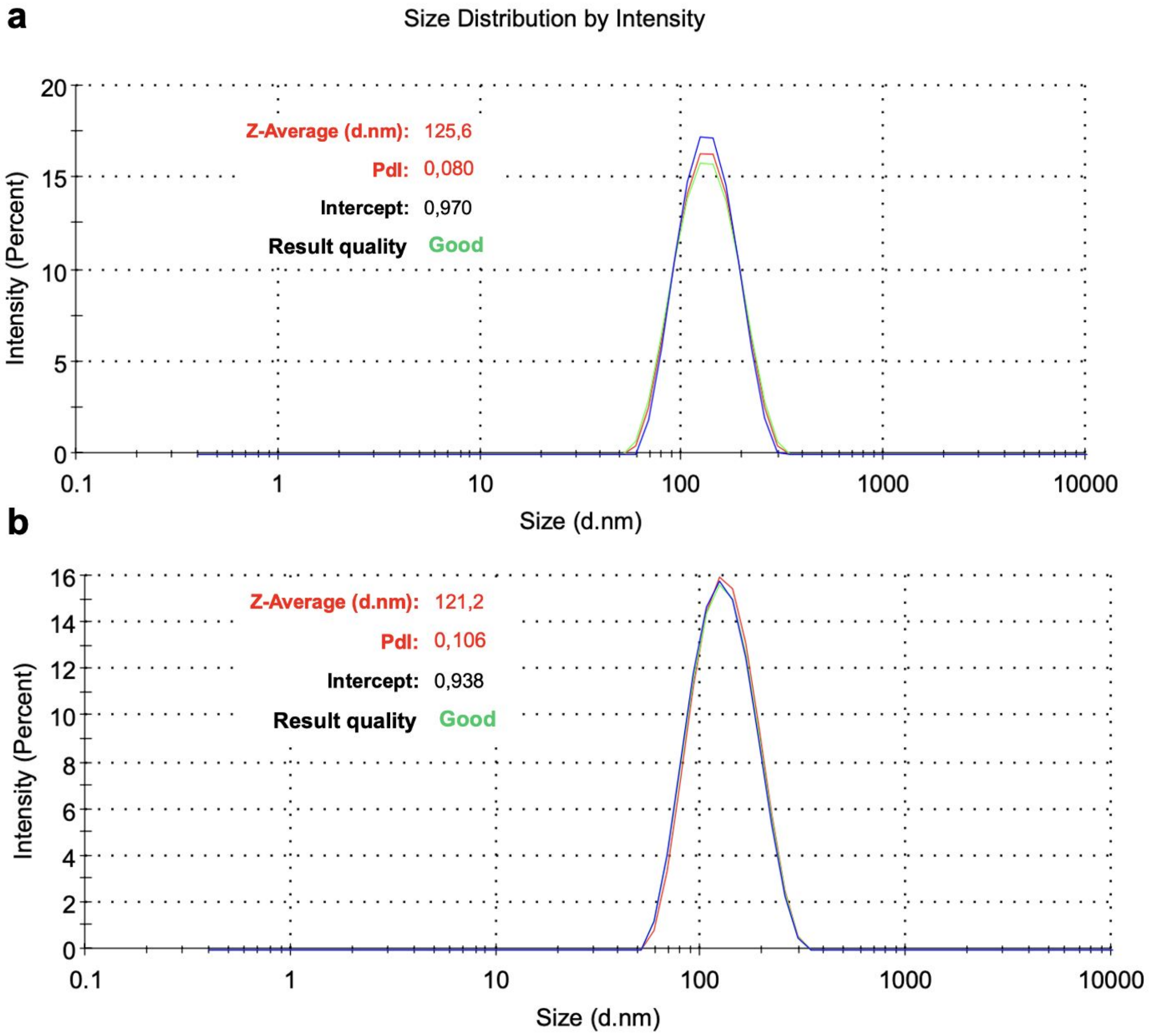

Figure S4. DLS (PMAA)-b-P(EG2MA-co-NIPAM) **(a), (b)** nanoparticles**.**

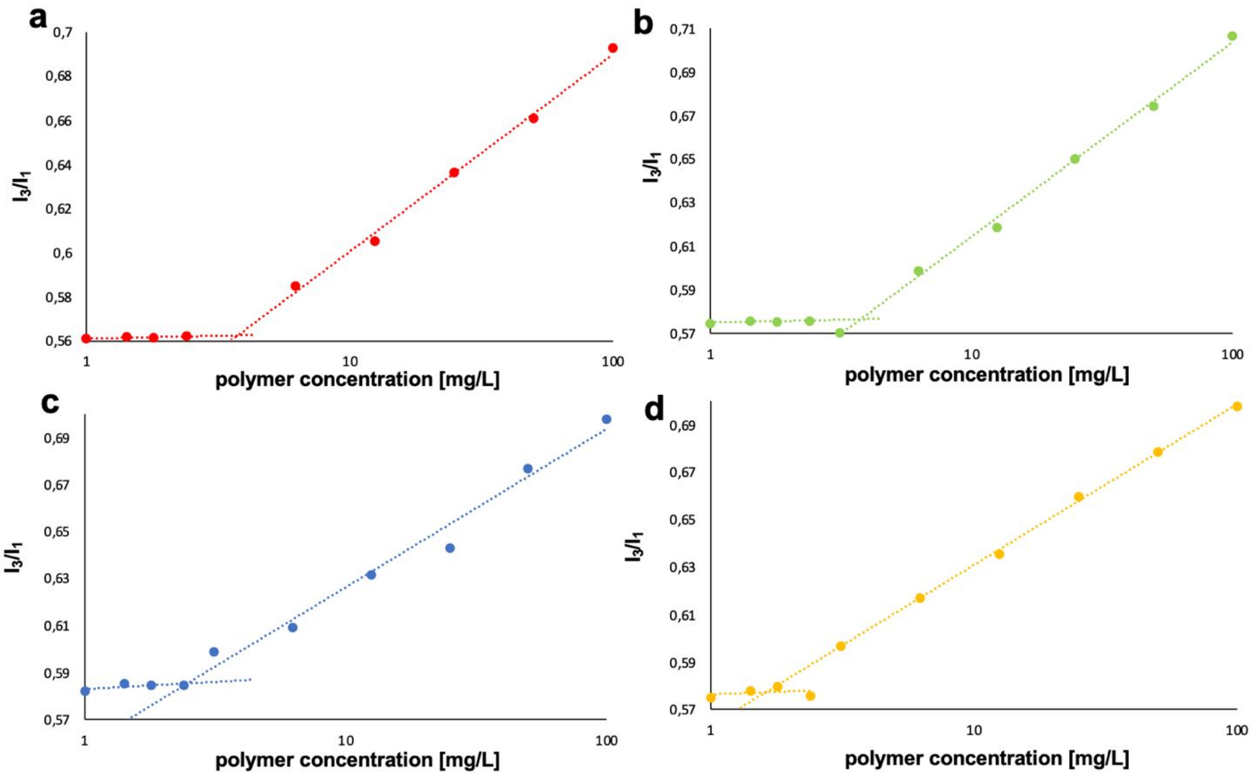

Figure S5. I3/I1 vs. polymer concentration showing the CMC of the final copolymer for **(a)** sample A (red) **(b)** sample B (green) **(c)** sample C (blue) **(d)** sample D (yellow).

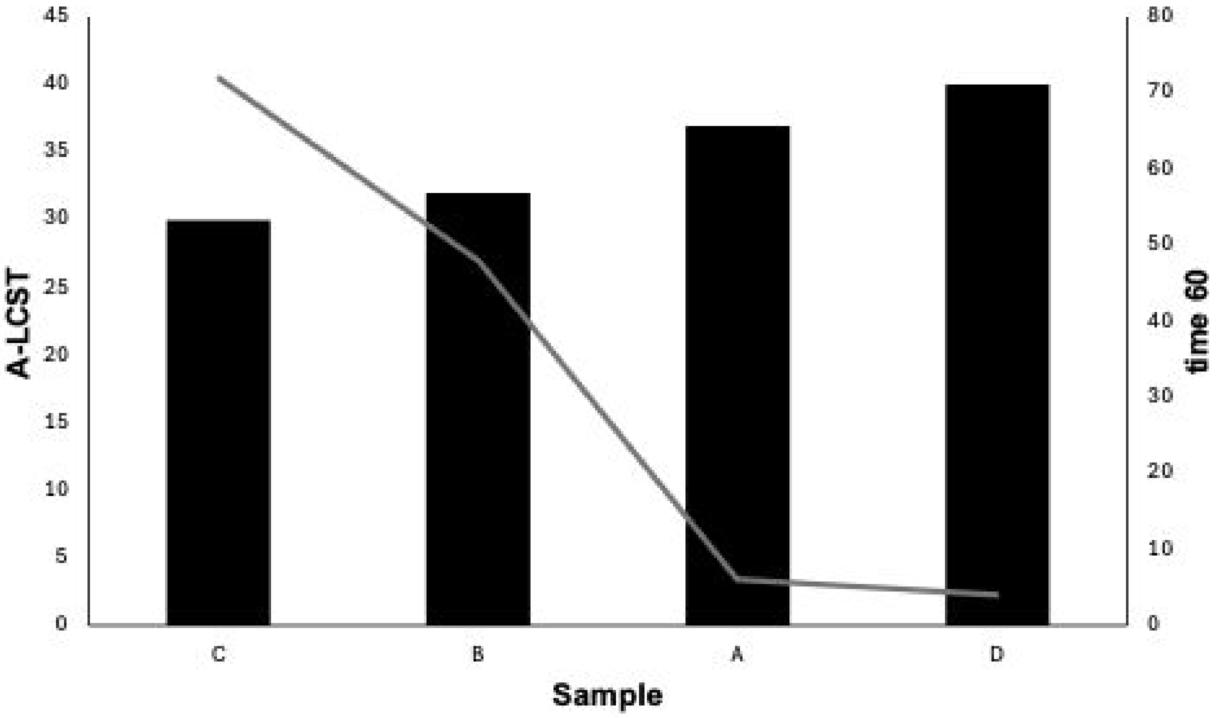

Figure S6. The bar plot represents the apparent LCST (°C) of formulations C, B, A, and D, while the grey line indicates the time required for FITC release to exceed 60% when exposed to an environment above the LCST. Increasing LCST values correspond to shorter release times, demonstrating the direct influence of polymer composition on the temperature-triggered diffusion rate.

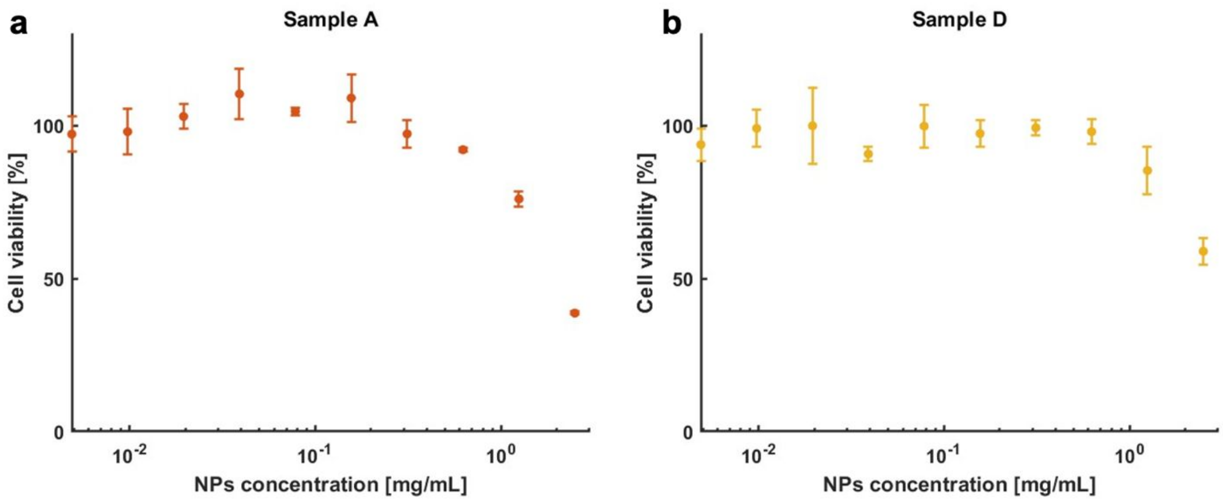

Figure S7. Percentage of cellular viability vs nanoparticle concentrations from in vitro biocompatibility test for **(a)** Sample A (red) and **(b)** Sample D (yellow).

#	Sample	5-FU Encapsulation Efficiency [%]	5-FU Drug Loading [%]	FITC Encapsulation Efficiency [%]	FITC Drug Loading [%]
A	**$(PMAA)_{50}$-b-$(EG_2MA_{17}$-co-$NIPAM_{33})$**	83.33 ± 2.04	2.84 ± 0.36	84.78 ± 2.01	3.28 ± 0.08
B	**$(PMAA)_{50}$-b-$(EG_2MA_{25}$-co-$NIPAM_{25})$**	84.62 ± 4.58	2.98 ± 0.41	86.87 ± 1.97	3.36 ± 0.08
C	**$(PMAA)_{50}$-b-$(EG_2MA_{46}$-co-$NIPAM_{4})$**	85.66 ± 4.65	3.84 ± 0.41	88.97 ± 1.65	3.44 ± 0.06
D	**$(PMAA)_{50}$-b-$(EG_2MA_{50}$-co-$NIPAM_{50})$**	91.02%± 5.63	2.87 ± 0.36	82.50 ± 2.88	3.38 ± 0.17

Table S1. Encapsulation efficiency (EE%) and drug loading (DL%) are reported in Table S1 for the different polymeric formulations.
